# Identification of 5P Chromosomes in Wheat-*Agropyron cristatum* Addition Line and Analysis of Its Effect on Homologous Pairing of Wheat Chromosomes

**DOI:** 10.3389/fpls.2022.844348

**Published:** 2022-02-24

**Authors:** Cuili Pan, Qingfeng Li, Haiming Han, Jinpeng Zhang, Shenghui Zhou, Xinming Yang, Xiuquan Li, Lihui Li, Weihua Liu

**Affiliations:** ^1^National Key Facility for Crop Gene Resources and Genetic Improvement, Institute of Crop Science, Chinese Academy of Agricultural Sciences, Beijing, China; ^2^School of Agriculture, Ningxia University, Yinchuan, China

**Keywords:** *Agropyron cristatum*, 5P chromosomes, molecular cytogenetics, homoeologous pairing, addition line

## Abstract

As an important wheat wild relative, the P genome of *Agropyron cristatum* (L.) Gaertn. (2*n* = 4*x* = 28) is very valuable for wheat improvement. A complete set of wheat-*A. cristatum* disomic addition lines is the basis for studying the genetic behavior of alien homoeologous chromosomes and exploring and utilizing the excellent genes. In this study, a wheat-*A. cristatum* derivative II-11-1 was proven to contain a pair of 5P chromosomes and a pair of 2P chromosomes with 42 wheat chromosomes by analyzing the fluorescence *in situ* hybridization (FISH) and expressed sequence tag (EST) markers. Additionally, cytological identification and field investigation showed that the 5P chromosome can weaken the homologous pairing of wheat chromosomes and promote the pairing between homoeologous chromosomes. This provides new materials for studying the mechanism of the alien gene affecting the homologous chromosome pairing and promoting the homoeologous pairing of wheat. In addition, chromosomal structural variants have been identified in the progeny of II-11-1. Therefore, the novel 5P addition line might be used as an important genetic material to widen the genetic resources of wheat.

## Introduction

Wheat (*Triticum aestivum* L., 2*n* = 42, AABBDD), as one of the most important food crops in the world, plays a significant role in ensuring food production and security. The homogenization of wheat varieties in major regions has narrowed their genetic background, which became the main bottleneck of breeding. Fortunately, there are plenty of beneficial genes in the wheat wild relatives that could be exploited and utilized for wheat improvement (Dong, 2000). The success of distant hybridization made it possible to transfer alien excellent genes to common wheat for creating new germplasms. Wheat disomic addition lines contain a pair of alien chromosomes in the wheat background, which is an important tool and bridge material for transferring alien excellent genes. Furthermore, they are favorable materials to identify the relationship between alien and wheat chromosomes, which could also be used for gene mapping. At present, most of the wheat wild relatives have been successfully hybridized with wheat, such as *Aegilops* (Gong et al., 2017; Du et al., 2019; Liu et al., 2019; Yi et al., 2019), *Secale cereal* L. (Li et al., 2016a,^2020^; Schneider et al., 2016; An et al., 2019), *Hordeum vulgare* L. (Szakacs and Molnal-Lang, 2010; Fang et al., 2014), *Haynaldia villosa* (Zhang et al., 2015a, 2018a,b), *Leymus racemosus* (Yang et al., 2017; Zhang et al., 2017a), and *Elytrigia repens* (Liu et al., 2017). Meanwhile, a large number of disomic addition lines and substitution lines were identified, which were used to create translocation lines and introgression lines containing desirable genes. Some introgression lines and translocation lines are widely used in wheat production. For instance, the cultivated variety Xiaoyan 6 was bred from the hybridization of wheat and *Thinopyrum* (Ma et al., 2018), and the crucial translocation lines T1RS⋅1BL and T6VS⋅6AL were from the hybridization of wheat and rye (Su et al., 2006) and *H. villosa* (Jiang et al., 2014; Gao et al., 2018), respectively.

*Agropyron cristatum* L. Gaertn. (2*n* = 28, PPPP) is an important wheat wild relative. It grows in arid grassland, hillside, hill, and desert and contains many desirable traits for wheat improvement, such as resistance to wheat leaf rust, powdery mildew, barley yellow dwarf, and wheat streak mosaic viruses (Dewey, 1984; Sharma et al., 1984; Ochoa et al., 2014) and tolerance to drought and low temperature (Limin and Fowler, 1987; Asay and Johnson, 1990; Dong et al., 1992), as well as with multiple spikelets and florets, small flag leaves, fertile tiller number and strong and tough stem (Dewey, 1984; Wu et al., 2006; Han et al., 2014; Jiang et al., 2018). The acquisition of wheat-*A. cristatum* disomic addition lines made it possible to utilize these desirable genes to improve wheat variety. This study has been committed to the hybridization of wheat and *A. cristatum* for a long time and has created a series of wheat-*A. cristatum* addition, translocation, and deletion lines successfully (Li and Dong, 1991, 1993; Li et al., 1995, 1997, 1998, 2016b; Luan et al., 2010; Song et al., 2013; Ye et al., 2015; Lu et al., 2016; Zhang et al., 2019). So far, wheat-*A. cristatum* 1P, 2P, 3P, 4P, 6P, and 7P disomic addition lines have been successfully created, and many excellent genes were located in specific chromosomes and transmitted into wheat (Wu et al., 2006; Han et al., 2014; Li et al., 2016a; Lu et al., 2016; Pan et al., 2017; Chen et al., 2018; Zhou et al., 2018). For instance, it has been found that *A. cristatum* 6P addition line carried gene clusters related to yield, such as multiple florets and grains per spike, and the 2P addition line possessed gene clusters related to disease resistance including powdery mildew, leaf rust, and stripe rust (Wu et al., 2006; Han et al., 2014; Li et al., 2016a). These genes were further mapped by creating translocation lines and introgression lines and developing specific markers for P chromosomes (Liu et al., 2010; Dai et al., 2012; Zhang et al., 2015b,c; Han et al., 2017, 2019; Zhou et al., 2018). However, wheat-*A. cristatum* 5P disomic addition line has not been obtained.

In this study, a wheat-*A. cristatum*-derived line II-11-1 with four *A. cristatum* chromosomes was used as a basic material for backcross and self-cross with recipient parent Fukuhokomugi (Fukuho). The purpose of this study was (1) to analyze the chromosome constitution of wheat-*A. cristatum*-derived line II-11-1, (2) to obtain wheat-*A. cristatum* 5P addition line, and (3) to analyze the effects of *A. cristatum* 5P chromosome on the chromosomes pairing. The obtained wheat-*A. cristatum* 5P addition line provided basic materials for further systematic study on genetic variation of wheat distant hybrid.

## Materials and Methods

### Materials

The plant materials included *T. aestivum* cv. Fukuho (2*n* = 6*x* = 42, AABBDD), *A. cristatum* accession Z559 (2*n* = 4*x* = 28, PPPP), wheat-*A. cristatum*-derived material II-11-1 (2*n* = 46) and other wheat-*A. cristatum* homoeologous group addition lines: II-3-1a (1P) (Pan et al., 2017), II-9-3 (2P) (Li et al., 2016a), 7365 (3P) (Zhou et al., 2018), II-21-2 (4P) (Liu et al., 2010), 4844-12 (6P) (Wu et al., 2006), and II-5-1 (7P) (Lu et al., 2016). All the above materials were provided by the Center of Crop Germplasm Resources Research at the Institute of Crop Science, Chinese Academy of Agricultural Sciences, Beijing, China.

### Observation on the Mitosis of Root Tip Cells and the Meiosis of Pollen Mother Cells (PMC)

The mitotic metaphase of root tip cells and the meiotic metaphase I of PMC of Fukuho, wheat-*A. cristatum*-derived material II-11-1, and the newly obtained 5P addition line were observed. Genomic *in situ* hybridization (GISH) was performed as described by Cuadrado et al. (2000), and the meiotic metaphase I of PMC was observed following the method of Jauhar and Peterson (2006).

### Fluorescence *in situ* Hybridization

The genomic DNA of *A. cristatum* accession Z559 and common wheat Fukuho were extracted using the cetyl trimethyl ammonium bromide (CTAB) method (Dellaporta et al., 1983). The P chromosome repetitive sequences, pAcTRT1 and pAcpCR2, were used as probes to identify the homologous groups of *A. cristatum* in II-11-1 and II-11-1b using the method described by Han et al. (2019). The barley clone pHvG38 contains the GAA-satellite sequence (Pedersen and Langridge, 1997), and the clone pAsl contains a 1 kb DNA repetitive sequence from *Aegilops tauschii* (Rayburn and Gill, 1986). The combination of pAs1 and pHvG38 allowed the discrimination of the three genomes in wheat. The procedure of fluorescence *in situ* hybridization (FISH) was carried out as described by Han et al. (2004) and Liu et al. (2010). Images were captured using an OLYMPUS AX80 fluorescence microscope (Olympus Corporation, Tokyo, Japan) equipped with a charge-coupled device (CCD) camera (Diagnostic Institute, Inc., Sterling Height, MI, USA) and then were processed with Photoshop CS 3.0.

### Molecular Marker Analysis

A total of 236 markers were used to identify the alien P chromatin and to determine its homoeologous group (Supplementary Table 1), of which 160 markers were described by Zhang et al. (2017b) and 76 markers were described by Li et al. (2016b). The PCR amplification procedure was performed as described by Luan et al. (2010). The amplified product was verified using 6% polyacrylamide gel electrophoresis (PAGE).

### Evaluation of the Agronomic Traits

All the tested materials were sown in a randomized complete block design with three replicates in the fields at Xinxiang (35°18′13.71″N, 113°55′15.05″E, Henan Province, China) during 2016–2017, 2017–2018, and 2018–2019 growing seasons. A total of 20 grains were evenly planted in 2.0 m rows spaced 0.3 m apart (Zhang et al., 2019). The agronomic traits were measured and quantified including grain number, spikelet number and kernel number per spikelet, thousand-grain weight, and effective tiller number. The Statistical Analysis System (version 9.2, SAS Institute, Cary, NC, USA) software was used for statistical analysis.

## Results and Analysis

### The Chromosome Composition Analysis of Wheat-*A. cristatum* II-11-1

**FIGURE 1:**
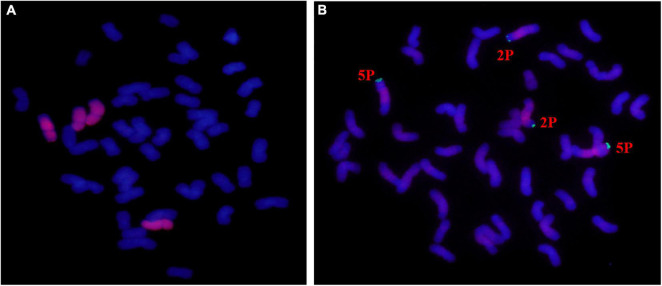
Mitosis genomic *in situ* hybridization (GISH)/fluorescence *in situ* hybridization (FISH) identification of II-11-1. **(A)** The whole-genome DNA probe of *Agropyron cristatum* was labeled as a red signal, and wheat chromosomes were restained as blue by DAPI. **(B)** The probes pAcTRT1 and pAcpCR2 were labeled as red and green, respectively, and wheat chromosomes were restained as blue by DAPI.

In the population composed of 50 individuals of II-11-1, 31 plants containing 42 wheat chromosomes and 4 *A. cristatum* chromosomes were identified by mitosis observation and GISH detection (Figure 1A). Additionally, pAcTRT1 and pAcpCR2 were used as probes to identify the additional chromosomes of *A. cristatum* in II-11-1. As shown in Figure 1B, the additional *A. cristatum* chromosomes were a pair of 2P and a pair of 5P according to the signal characteristics. So, the derivative II-11-1 (2*n* = 46) was preliminarily determined to be a wheat-*A. cristatum* 2P and 5P disomic addition line.

### Identification of II-11-1 With Expressed Sequence Tag (EST)-STS Markers

**FIGURE 2:**
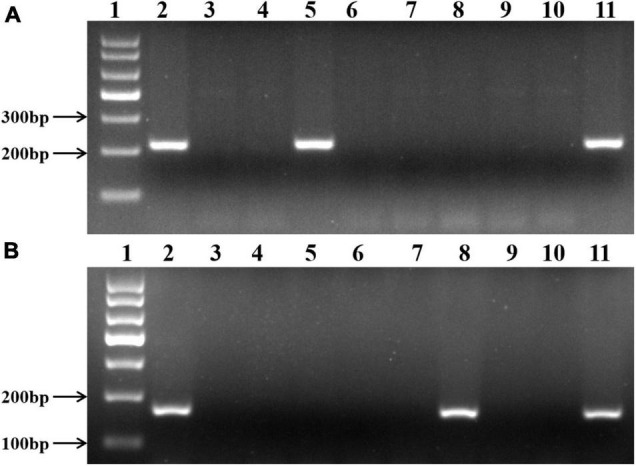
Molecular marker identification of II-11-1. **(A,B)** 2P chromosome-specific marker, Agc3725, and 5P chromosome-specific marker, Agc737, respectively. 1: Puc19 DNA MSP/I HPA II marker; 2: *A. cristatum* Z559; 3: common wheat Fukuho; 4: Wheat-*A. cristatum* 1P addition line II-3-1a; 5: Wheat-*A. cristatum* 2P addition line II-9-3; 6: Wheat-*A. cristatum* 3P addition line 7365; 7: Wheat-*A. cristatum* 4P addition line II-21-2; 8: Wheat-*A. cristatum* 5P addition line II-11-1b; 9: Wheat-*A. cristatum* 6P addition line 4844-12; 10: Wheat-*A. cristatum* 7P addition line II-5-1; 11: Wheat-*A. cristatum* derivation line II-11-1.

To further confirm the identity of *A. cristatum* chromosomes in II-11-1, the EST-STS markers specific to the 2 and 5 homoeologous groups were employed to identify II-11-1 and wheat-*A. cristatum* disomic addition lines (1P, 2P, 3P 4P, 6P, and 7P). Results showed that 36 pairs of 2P chromosome-specific primers amplified specific bands for the wheat-*A. cristatum* 2P addition line and II-11-1 (Figure 2A) and 78 pairs of 5P chromosome-specific markers amplified specifically in II-11-1 and II-11-1b (Figure 2B and Supplementary Table 2). Therefore, it was further confirmed that II-11-1 contains 2P and 5P chromosomes, which could be used as a basic material for the separation and identification of wheat-*A. cristatum* 5P addition line.

### Chromosome Behavior Analysis of II-11-1 During Meiosis

**FIGURE 3:**
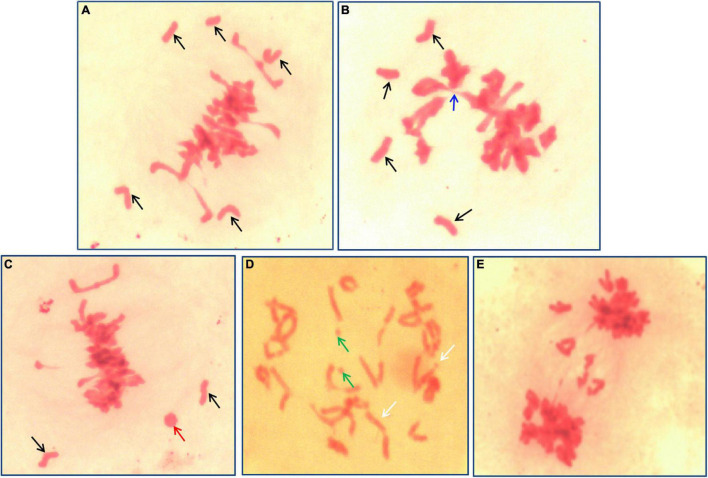
Meiosis identification of II-11-1. **(A–D)** Meiosis metaphase I; **(E)** meiosis anaphase I, chromosome bridges, and lagging chromosomes. Black arrows refer to univalents, blue arrows to multivalents, green arrows to chromosome fragments, and red arrows to ring chromosomes.

Meiosis pairing was observed in PMCs to analyze the chromosome behavior of II-11-1 during the generation of the gamete. Results indicated that there existed univalent and multivalent chromosomes, chromosome fragments, and ring chromosomes at metaphase (Figures 3A–D). Meanwhile, chromosome lagging and chromosome bridge were found at anaphase (Figure 3E). The chromosome configuration statistics of II-11-1 at metaphase showed that the average univalents, rod bivalents, ring bivalents, trivalents, quadrivalents and chromosome fragments were 3.95, 3.81, 16.85, 0.16, 0.08, and 0.18, respectively (Table 1). The above results indicated that the behaviors of chromosome pairing were abnormal during meiosis in II-11-1.

**TABLE 1:** Pollen mother cells (PMC) meiosis metaphase I chromosome configuration of wheat-*Agropyron cristatum* derivatives II-11-1 and 5P addition line II-11-1b.

Materials	2n	Number of cells	Chromosome configuration						
			Univalents	Bivalent			Trivalent	Quadrivalent	Fragment
				Rod	Ring	Total			
II-11-1	46	74	3.95 (0–8)	3.81 (1–8)	16.85 (11–19)	20.66 (18–22)	0.18 (0–1)	0.08 (0–1)	0.18 (0–2)
II-11-1b	44	71	2.08 (0–6)	2.72 (0–8)	17.56 (11–22)	20.28 (18–22)	0.23 (0–1)	0.13 (0–1)	0.14 (0–1)
Fukuho	42	50	0.06 (0–2)	1.95 (1–4)	19.32 (17–21)	20.97 (20–21)	–	–	–

### Identification of the II-11-1 Progenies by Molecular Markers

The markers specific to 2P and 5P chromosomes were used to detect the BC_1_F_2_ population of II-11-1 with Fukuho as a recurrent parent. According to the results, 212 BC_1_F_2_ individuals were divided into four types, namely, type I, type II, type III, and type IV. There were 47 plants (21.76%) with 5P chromosome only in type I, 54 plants (25.00%) with 2P chromosome only in type II, 66 plants (30.56%) with both 2P and 5P chromosomes in type III, and 45 plants (20.83%) without P chromosomes in type IV. Among them, the type I individuals with the 5P chromosome only provided the candidate materials for further identifying the wheat-*A. cristatum* 5P addition line.

### Identification and Molecular Cytological Detection of Wheat-*A. cristatum* 5P Addition Line

**FIGURE 4:**
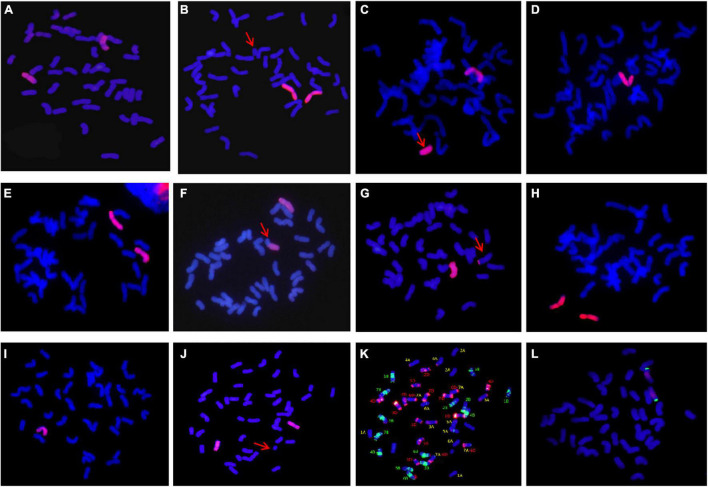
Mitosis GISH/FISH identification of II-11-1b. **(A–J)** *A. cristatum* genome DNA probe was labeled as a red signal; **(K)** pAs1 and pHvG38 were labeled as red and green, respectively; **(L)** pAcTRT1 and pAcpCR2 were labeled as red and green, respectively, and wheat chromosomes were restained as blue by DAPI.

The chromosomal composition of 47 candidate individual plants in type I was further identified using GISH using the root tips (Table 2). Statistical results showed that there were 15 plants with 44 chromosomes, of which 12 plants were composed of 42 wheat chromosomes and 2 *A. cristatum* chromosomes (Figure 4A); 2 plants composed of 41 wheat chromosomes, 1 wheat telomere, and 2 *A. cristatum* chromosomes (Figure 4B) and 1 plant consisted of 42 wheat chromosomes, 1 *A. cristatum* telomere, and 1 *A. cristatum* chromosome (Figure 4C). There were 26 plants with 43 chromosomes, of which 22 plants were composed of 42 wheat chromosomes and 1 *A. cristatum* chromosome (Figure 4D); 2 plants contained 41 wheat chromosomes and 2 A. cristatum chromosomes (Figure 4E); 1 plant was composed of 41 wheat chromosomes, 1 whole arm translocation, and 1 A. cristatum chromosome (Figure 4F); 1 plant consisted of 41 wheat chromosomes, 1 small alien segment translocation, and 1 A. cristatum chromosome (Figure 4G). There were 5 plants with 42 chromosomes, of which 3 plants consisted of 40 wheat chromosomes and 2 A. cristatum chromosomes (Figure 4H) and 2 plants consisted of 41 wheat chromosomes and 1 A. cristatum chromosome (Figure 4I). One plant consists of 45 chromosomes with 42 wheat chromosomes, 1 wheat telomere, and 2 A. cristatum chromosomes (Figure 4J). Combined with the results of FISH identification, we found that 6D-7A translocation occurred in wheat chromosomes (Figure 4KSupplementary Figure 1A). Among the 47 individuals of type I, the 12 plants with 42 wheat and 2 A. cristatum chromosomes were further identified using FISH with the pAcTRT1 and pAcpCR2 probes. It was also revealed that the pair of A. cristatum chromosomes was 5P (Figure 4L and Supplementary Figure 1B). Finally, they were identified as a novel wheat-A. cristatum 5P addition line and named as II-11-1b.

**TABLE 2:** Chromosomal constitutions of 47 plants derived from plant II-11-1b.

No. of plants	No. of chromosomes	No. of plants	Constitution	Example
15	2n = 44	12	42W + 2A	Figure 4A
		2	41W + 1t^W^ + 2A	Figure 4B
		1	42W + 1A + 1t^A^	Figure 4C
26	2n = 43	22	42W + 1A	Figure 4D
		2	41W + 2A	Figure 4E
		1	41W + 1W.A + 1A	Figure 4F
		1	41W + 1W-A + 1A	Figure 4G
5	2n = 42	3	40W + 2A	Figure 4H
		2	41W + 1A	Figure 4I
1	2n = 45	1	42W + 2A + 1t^W^	Figure 4J

*W, wheat chromosomes; A, A. cristatum chromosomes; t, telosome; W.A, Robertsonian translocation; W-A, non-Robertsonian translocation.*

### Meiosis Abnormality and 5P Chromosome Functional Analysis

**FIGURE 5:**
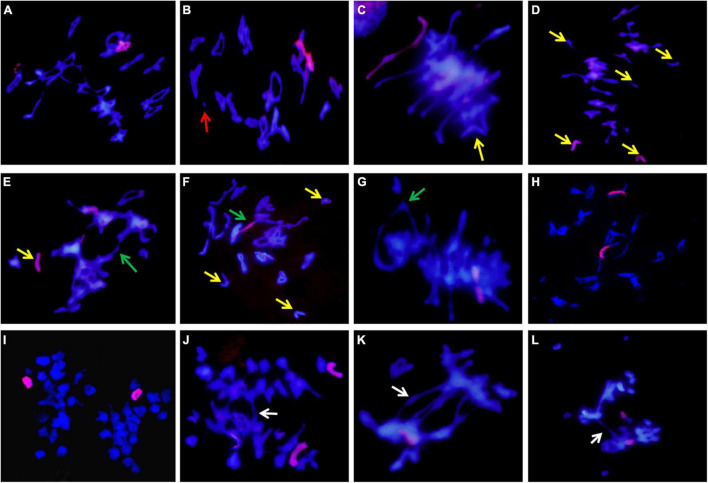
Meiosis GISH identification of II-11-1b. **(A–L)** *A. cristatum* genome DNA probe was labeled as a red signal; wheat chromosome restained blue by DAPI. **(A–H)** Meiotic metaphase I; **(I–L)** anaphase I. The red arrows refer to chromosome fragments, the yellow arrows refer to univalents, the green arrows refer to polyvalents, and the white arrows refer to chromosome adhesion or chromosome bridge.

The meiosis metaphase I chromosome configuration of II-11-1b was observed and counted (Table 1). Except for the normal bivalent formation at metaphase (Figure 5A) and normal separation at anaphase (Figure 5I), chromosome behavioral abnormalities were also identified (Figures 5B–H,J–L). For example, there were chromosome fragments (Figure 5B), univalents of wheat chromosomes (Figure 5C) and *A. cristatum* 5P chromosomes (Figure 5D), multivalents, including trivalent and tetravalent, formed by wheat chromosomes only or wheat and *A. cristatum* 5P chromosomes (Figures 5E–G). In addition, the chromosome bridge and the division desynchrony with different numbers of lagging chromosomes were also observed (Figures 5H–L). Statistical analysis of the meiosis metaphase I showed that the 5P addition line II-11-1b had significantly higher frequencies of univalents and multivalents compared with the parent Fukuho (Table 1). The presence of multivalents indicates the existence of some homoeologous chromosome synapses or chromosomal rearrangement. At the anaphase of meiosis, the loss of univalents and the abnormal segregation of multivalents can cause changes in the chromosomal composition of their progenies. Thus, it is predicted that the chromosome 5P of *A. cristatum* has the function of inducing chromosome breakage, promoting synapses of the homoeologous chromosome.

### Evaluation of Agronomic Traits

**FIGURE 6:**
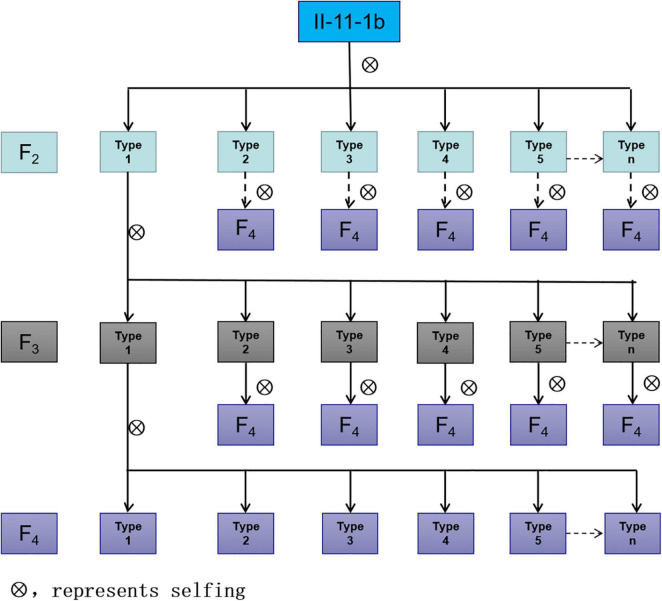
The pattern diagram of segregation of self-crossing progenies of II-11-1b.

**FIGURE 7:**
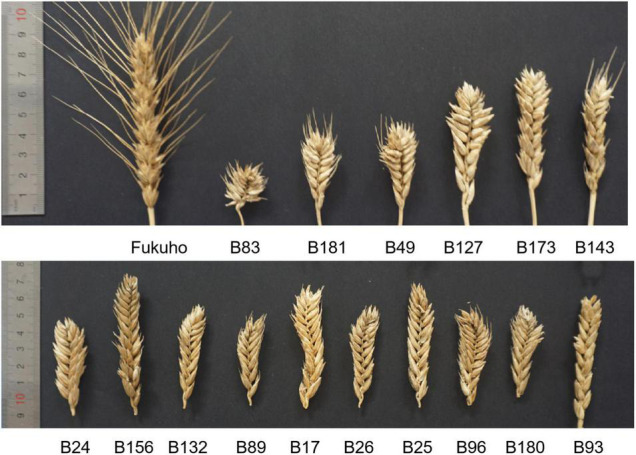
Segregation of spike traits was detected in the F_2_ population. Fukuho: parent; F_2_: B83, B181, B49, B127, B173, B143, B24, B156, B132, B89, B17, B26, B25, B96, B180, B93.

According to the results of observation, statistics, and evaluation in three growing seasons, II-11-1b progenies with 5P chromosomes showed segregation in agronomic traits (Type 1 in Figure 6), and progenies without 5P chromosomes gradually stabilized (Types 2-n in Figure 6). For example, different types of spike traits were detected in II-11-1b progenies with 5P chromosomes (Figure 7). It showed that 5P chromosomes resulted in a decrease in fertility, which was reflected in the significant decrease in the kernel number per spikelet and per spike and the significant increase in the number of sterile florets (*p* < 0.05) in 5P positive plants compared with negative plants (Table 3). Meanwhile, the variation (statistical standard deviation) in tiller number, plant height, spike length, etc., of 5P positive plants were higher than those of negative plants (Table 3). Combined with the results of traits and molecular cytology, it is speculated that the presence of 5P chromosomes might influence the genetic stability by regulating the homologous and homoeologous chromosome pairing behavior during meiosis, thus leading to fertility decrease and trait separation in the progenies.

**TABLE 3:** Agronomic traits of II-11-1b progenies.

Year	Type	Tillering number	Plant height (cm)	Spike length (cm)	Spikelet number	Spikelet density	Under-spike internodal ratio	Kernel number per spikelet	Kernel number per spike	Number of sterile floret
2016-2017	5P +	12.90b ± 9.77 (3-37)	80.84b ± 9.64 (62.0-98.0)	8.60a ± 1.13 (4.7-9.5)	18.29a ± 2.17 (15-23)	20.19a ± 4.18 (16.09-38.29)	0.31b ± 0.08 (0.15-0.39)	3.69b ± 0.94 (1-4)	43.25b ± 12.31 (0-68)	9.53a ± 5.75 (1-24)
	5P –	17.96a ± 3.59 (9-28)	85.99a ± 4.91 (79.0-90.5)	8.92a ± 0.68 (7.1-11.0)	18.49a ± 1.81 (16-21)	19.79a ± 0.91 (17.78-24.84)	0.35a ± 0.03 (0.29-0.40)	4.49a ± 0.52 (3-5)	56.25a ± 7.61 (48-80)	4.50b ± 0.49 (0-6)
2017-2018	5P +	10.23b ± 10.02 (3-40)	86.75a ± 8.98 (59.0-98.5)	8.81a ± 0.98 (7.1-12.0)	17.61b ± 2.45 (14-23)	18.85b ± 4.73 (15.79-35.01)	0.33b ± 0.09 (0.16-0.39)	3.78b ± 0.88 (1-4)	43.15b ± 12.53 (0-69)	7.53a ± 6.31 (1-29)
	5P –	13.63a ± 3.43 (7-27)	90.35a ± 5.03 (86.0-107.0)	8.96a ± 0.57 (7.8-10.5)	18.91a ± 1.79 (15-20)	19.99a ± 0.97 (17.21-21.24)	0.37a ± 0.03 (0.29-0.40)	4.36a ± 0.49 (3-5)	58.78a ± 6.77 (44-82)	3.38b ± 0.41 (1-6)
2018-2019	5P +	12.63b ± 9.43 (4-35)	82.34a ± 9.35 (61-97.5)	8.84a ± 1.07 (6.7-10.4)	18.04a ± 2.77 (14-24)	20.07a ± 4.77 (16.53-37.65)	0.31b ± 0.07 (0.15-0.37)	3.65b ± 0.97 (1-4)	41.04b ± 12.17 (3-77)	8.33a ± 4.55 (1-24)
	5P –	16.57a ± 9.77 (6-28)	85.26a ± 5.77 (69-89.5)	9.20a ± 0.77 (6.8-11.5)	18.25a ± 1.76 (16-21)	20.22a ± 0.87 (17.83-23.37)	0.33a ± 0.03 (0.28-0.38)	4.17a ± 9.77 (3-5)	53.01a ± 5.67 (42-77)	3.58b ± 0.37 (0-6)

*5P+ represents plants with 5P chromosomes and 5P- represents plants without 5P chromosomes. Means followed by different letters (“a” and “b”) indicate the significant differences at 0.05 level.*

## Discussion

### The Significance of Wheat-*Agropyron cristatum* 5P Addition Line

Wheat wild relatives as gene resource pools provide abundant genetic resources for wheat improvement. Lots of alien genes of wild relatives have been introgressed into wheat by distant hybridization, which shows great potential in improving yield and quality, disease resistance, and stress resistance. Among the progenies of distant hybridization, wheat-alien disomic addition lines played a very important role in the transfer of alien excellent genes. A complete set of wheat-alien disomic addition lines is an important genetic material for studying the genetic relationship, origin and evolution of species, gene expression, and interaction of chromosomes. To fully explore the desirable genes in *A. cristatum*, it is necessary to establish a complete wheat-*A. cristatum* addition line. In the previous studies, all the wheat-*A. cristatum* addition lines except 5P have been obtained. In this study, we identified the presence of 5P chromosomes in II-11-1. By further backcrossing and selfing, the wheat-*A. cristatum* 5P addition line was identified. So far, we have obtained a complete set of wheat-*A. cristatum* addition lines (1P–7P), which provided materials for the systematic study of the excellent exogenous genes from *A. cristatum*. However, we found that as long as there are 5P chromosomes, there will always be meiosis abnormality and trait separation in the progenies (Table 3). This increases the difficulty in the identification and preservation of the wheat-*A. cristatum* 5P addition line and also explains its unavailability for a long time. In addition, the 5P addition line is an excellent material for studying the abnormal behavior of chromosomes during meiosis.

### The Role and Value of 5P Chromosome: Induced Homoeologous Recombination

To date, some plentiful wild relatives have been successfully hybridized with wheat. However, due to the presence of genes that control homologous chromosome pairing (such as the *Ph1* gene on the 5B chromosome), chromosome pairing is difficult to occur between wheat and its wild relatives. This mechanism not only ensures the genetic stability of wheat but also restricts the chromosome recombination and the exogenous gene transfer between wheat and its wild relatives, thus hindering the application of excellent genes in breeding. The discovery of genes that inhibit homologous chromosome pairing has laid a foundation for the transfer of exogenous excellent genes and the creation of new germplasm resources (Ceoloni and Donini, 1993). At present, the *Ph* suppressor gene has been found in several wild relatives of wheat. For example, the 5Mg chromosome of *Aegilops geniculata* contained genes that promote synapsis and crossing in prophase I of meiosis in wheat (Tiwari et al., 2015; Koo et al., 2017). *Aegilops speltoides* 5S chromosome contained a QTL (*QPh.ucd-5S*) that could increase homeologous chromosome pairing and regulate recombination between homologous chromosomes in *T. aestivum* × *Ae. speltoides* hybrids (Dvorak et al., 2006). Genes that affect chromosome pairing during meiosis were also found in the 5U chromosome of *Aegilops umbrella* (Riley et al., 1973), 4M*^g^* chromosome of *A. geniculata* (Kynast et al., 2000), and 3S chromosome of *A. speltoides Tausch* (Li et al., 2019).

The offspring of distant hybridization might exhibit abnormal chromosome pairing behavior during meiosis. Observing the chromosomal configurations during meiosis of the alien addition line is a crucial way to analyze its stability. Based on the previous studies, it is speculated that there may also exist genes affecting homologous pairing in *A. cristatum*. For instance, by evaluating the *Ph*-suppressing effect of P chromosomes (1P–6P) and deletion *ph1b* of the *Ph1* gene, it showed that they all displayed a significantly higher level of homoeologous pairing than the control except for 2PL and 2PS, but allosyndetic associations between P and ABD genomes were very rare, which had no prospect in the transfer of alien genes (Jubault et al., 2006). The results of the 5P addition line were inconsistent with this study, which may be due to the different sources of *A. cristatum*. In this study, the theoretical chromosomal configurations of II-11-1 and II-11-1b should be 21 wheat chromosomes bivalents plus 2 *A. cristatum* bivalents and 21 wheat chromosomes bivalents plus 1 *A. cristatum* bivalent, respectively. However, the statistical results showed that the ratio of univalents in II-11-1 and II-11-1b was 9.85 and 2.08, respectively, trivalents was 0.18 and 0.23, respectively, and quadrivalents was 0.08 and 0.13 respectively (Table 1). Meanwhile, the wheat Fukuho had a significantly low univalents ratio of 0.06, and there is no trivalent or quadrivalent. In the anaphase II and telophase II of meiosis, there also existed an abnormal phenomenon including fragments, bridges, lagging chromosomes, and the chromosome adhesions in II-11-1 and II-11-1b. In the progenies of II-11-1b, wheat-*A. cristatum* 5P translocation lines were identified (Figures 4F–G). Thus, it is supposed that the 5P chromosome might play a comprehensive and complex role in regulating chromosomal behavior during meiosis, including the inhibition of the *Ph* gene to promote the synapses of the homoeologous chromosome, and the function similar to the gametocidal chromosome, which induces chromosome breakage and recombination and promotes the formation of chromosomal translocation in the progenies.

Meiotic homoeologous recombination could facilitate gene introgression to diversify the wheat genome for germplasm development. Therefore, the wheat-*A. cristatum* 5P addition line II-11-1b is a potential and valuable material for gene introgression and gene mapping based on recombination between homoeologous chromosomes in wheat. The studies of the 5P chromosome further enhance our understanding of the wheat genome and its homoeologous counterparts *A. cristatum* and expand the genetic variability of the wheat genome.

## Data Availability Statement

The original contributions presented in the study are included in the article/Supplementary Material, further inquiries can be directed to the corresponding authors.

## Author Contributions

WL and LL conceived the research. CP and QL performed the research and wrote the manuscript. CP, QL, HH, JZ, SZ, XY, and XL participated in the preparation of both the reagents and materials. All authors contributed to the article and approved the submitted version.

## Conflict of Interest

The authors declare that the research was conducted in the absence of any commercial or financial relationships that could be construed as a potential conflict of interest.

## Publisher’s Note

All claims expressed in this article are solely those of the authors and do not necessarily represent those of their affiliated organizations, or those of the publisher, the editors and the reviewers. Any product that may be evaluated in this article, or claim that may be made by its manufacturer, is not guaranteed or endorsed by the publisher.
