# Supplementary material for: Identification of 5P Chromosomes in Wheat-Agropyron cristatum Addition Line and Analysis of Its Effect on Homologous Pairing of Wheat Chromosomes
Source: Front Plant Sci. 2022 Feb 24;13:844348. doi: 10.3389/fpls.2022.844348 (PMC8908377; doi:10.3389/fpls.2022.844348)
Supplement: Supplementary file 1 [file Data_Sheet_1.docx]

Supplementary Material

Table S1 The lists of all primers in this study

| No. | **Unigene ID** | **Homoeologous group** | **Reference** | **Forward primer** | **Forward primer sequence** | **Reverse primer** | **Reverse primer sequence** | ***T*_m_** (°C) |
| --- | --- | --- | --- | --- | --- | --- | --- | --- |
| 1 | Unigene9023_All | 5P | Zhang et al. 2017 | AgC9023F | AGGCCCCGATAGCAGCAA | AgC9023R | CGTTGCTGCTGTTGTCGTCA | 59 |
| 2 | Unigene10395_All | 5P | Zhang et al. 2017 | AgC10395F | TAGAGATCTTGGTCCCGACA | AgC10395R | TAGAGGGCTGGGCTCTAACA | 59 |
| 3 | Unigene11668_All | 5P | Zhang et al. 2017 | AgC11668F | GGAAGGCTAGCATCGTTCTG | AgC11668R | CAGGATTCGGAAAAAGTCCA | 59 |
| 4 | Unigene21114_All | 5P | Zhang et al. 2017 | AgC21114F | TAAGATCTCCCCGGTAGCTG | AgC21114R | TCTGAAGTATTTGTTTGCTGCAT | 59 |
| 5 | Unigene32371_All | 5P | Zhang et al. 2017 | AgC32371F | GTTCCTCGCTACTGCTAGGC | AgC32371R | TTTCGGGTCTTGCTTCAGTT | 59 |
| 6 | Unigene10097_All | 5P | Zhang et al. 2017 | AgC10097F | AGTCGCGTCGAGAAAACAAC | AgC10097R | ACAGCTGTTTGTGCTTGTGG | 59 |
| 7 | Unigene21866_All | 5P | Zhang et al. 2017 | AgC21866F | TGTACCCTGACGAGCTGCCA | AgC21866R | CCGATTCTGCTGCGTGTTC | 59 |
| 8 | Unigene22121_All | 5P | Zhang et al. 2017 | AgC22121F | TTCCATATGATGAAGCCCCA | AgC22121R | AAGCACCTCAGACTGGTAGCAC | 59 |
| 9 | Unigene49470_All | 5P | Zhang et al. 2017 | AgC49470F | TGAAGTTCAGGTGTGGCGTA | AgC49470R | CTCTAGGCCTCTCCGCTGT | 59 |
| 10 | Unigene2175_All | 5P | Zhang et al. 2017 | AgC2175F | TGTTTCCCCAAGTTTTTAGCA | AgC2175R | AGATCCTTGCATCGGTTCTG | 59 |
| 11 | Unigene32810_All | 5P | Zhang et al. 2017 | AgC32810F | TACTGACCTGATGCCGACAA | AgC32810R | ATATGCCAAGGGCCTTCTCT | 59 |
| 12 | Unigene34035_All | 5P | Zhang et al. 2017 | AgC34035F | AGAACAGATGCACAACAAATC | AgC34035R | CATATATGTCAATGAAACTCCC | 59 |
| 13 | Unigene4598_All | 5P | Zhang et al. 2017 | AgC54598F | TACGGTGATAGGTGCGATGA | AgC54598R | GTCCGAGCTCTTGAGTGTGA | 59 |
| 14 | Unigene71979_All | 5P | Zhang et al. 2017 | AgC71979F | CTTCCAGATGTGCAGCAAAA | AgC71979R | CATTCCACCGAGTTGGTTCT | 59 |
| 15 | Unigene32606_All | 5P | Zhang et al. 2017 | AgC32606F | ATCTTGGCTAGGGCGGTCTT | AgC32606R | AGTACACGCAGATGGTGCTCTC | 59 |
| 16 | Unigene26558_All | 5P | Zhang et al. 2017 | AgC26558F | CCTGGTGTGTTGTTCGTCAG | AgC26558R | AGCCTCAGGTTCAGCATCTC | 59 |
| 17 | Unigene9932_All | 5P | Zhang et al. 2017 | AgC9932F | CACCATCCTTCTTCTTTTTTGGA | AgC9932R | AAAAAGAGGTTCCGGCAACA | 59 |
| 18 | Unigene27139_All | 5P | Zhang et al. 2017 | AgC27139F | TGTCCAGAGTGAATGGCTGC | AgC27139R | CGGCAGGTGCAAATAGCA | 59 |
| 19 | Unigene615_All | 5P | Zhang et al. 2017 | AgC615F | GCAGGCTGAAGAGGTTGTTC | AgC615R | GAGGAGCCACAAGGAAACTG | 59 |
| 20 | Unigene2378_All | 5P | Zhang et al. 2017 | AgC2378F | CTGACCTGTGTTGTCGCAGT | AgC2378R | AAAGCCTTGCATGAATCACC | 59 |
| 21 | Unigene13319_All | 5P | Zhang et al. 2017 | AgC13319F | AGATGAGGCGCTGCTTGTAT | AgC13319R | TCATGTTTGCTGGCAAGAAG | 59 |
| 22 | Unigene34306_All | 5P | Zhang et al. 2017 | AgC34306F | CGGATAACAAGAAACAATTCGC | AgC34306R | TGTTTGTCATCCTTGTGTTGCAC | 59 |
| 23 | Unigene4457_All | 5P | Zhang et al. 2017 | AgC4457F | GCAAACTGGATTAGTGCCTTCA | AgC4457R | AGCTTTTCCAACTCATCTTGCT | 59 |
| 24 | Unigene72356_All | 5P | Zhang et al. 2017 | AgC72356F | GAACCTGGAGAGCAATCAGC | AgC72356R | CAATGGCAAGGCAGTATGTG | 59 |
| 25 | Unigene65939_All | 5P | Zhang et al. 2017 | AgC65939F | TTTCTCTGTCCACCTTCGCT | AgC65939R | GACAAGGACCTCATGCCATT | 59 |
| 26 | Unigene25041_All | 5P | Zhang et al. 2017 | AgC25041F | GCAAGATAAGCTCCACACCA | AgC25041R | CGAGACTTGAGCATGAACCA | 59 |
| 27 | Unigene39273_All | 5P | Zhang et al. 2017 | AgC39273F | ATTGATGGACAGCACGAACG | AgC39273R | TTGCTCTTGGTGATCGGATC | 59 |
| 28 | Unigene39998_All | 5P | Zhang et al. 2017 | AgC39998F | CAGGTTCCAAGCCATTCAGT | AgC39998R | CTGATGTGGATGACTGGACAA | 59 |
| 29 | Unigene22373_All | 5P | Zhang et al. 2017 | AgC22373F | AATCAAGGTACTCCAATCCTCGT | AgC22373R | GGAGAGAATTTCTGCAAAGGGA | 59 |
| 30 | Unigene5771_All | 5P | Zhang et al. 2017 | AgC5771F | CTGAAGGAAGAAGCCCACTG | AgC5771R | CTGCACCAGGGAAATGTCTT | 59 |
| 31 | Unigene822_All | 5P | Zhang et al. 2017 | AgC822F | CTTGGCAAAATGATCTCTTG | AgC822R | TCCACACTTCAGCATTTTTC | 59 |
| 32 | Unigene22557_All | 5P | Zhang et al. 2017 | AgC22557F | CACAGTATCTCCGGGTCCAC | AgC22557R | TCTACCACCAGTCCACCACA | 59 |
| 33 | Unigene71658_All | 5P | Zhang et al. 2017 | AgC71658F | TCACAATCGTGTCCCAGGTA | AgC71658R | TTTATTTCTCCCTGATGGCG | 59 |
| 34 | Unigene25666_All | 5P | Zhang et al. 2017 | AgC25666F | GCAATTGAAGTGAAACTCTTCCC | AgC25666R | TGCTTCTGCAATCACGGCTA | 59 |
| 35 | Unigene29276_All | 5P | Zhang et al. 2017 | AgC29276F | CGTCATCGGTACCAAATTCC | AgC29276R | TAAGGTTTCCCGTTGAAGGA | 59 |
| 36 | Unigene35699_All | 5P | Zhang et al. 2017 | AgC35699F | CTGTAATGCACAGAATTGCA | AgC35699R | TTGCTACTACTGTGTTGGTCC | 59 |
| 37 | Unigene9229_All | 5P | Zhang et al. 2017 | AgC9229F | AACAACTGTCTCGCGGATCT | AgC9229R | AGTTGTGCCATCCTGTAGGG | 59 |
| 38 | Unigene24612_All | 5P | Zhang et al. 2017 | AgC24612F | AGGAGATGTGGCATAGACTCAGTT | AgC24612R | AGCGTTACGGTGTTTCCACA | 59 |
| 39 | Unigene4764_All | 5P | Zhang et al. 2017 | AgC4764F | GCTGGGTGTTGAGATGGGA | AgC4764R | TGAAGATAGCCAAACGGCATAG | 59 |
| 40 | Unigene5477_All | 5P | Zhang et al. 2017 | AgC5477F | TGCGAGCATCGACCTTTAGT | AgC5477R | GAGGGCATAAATTCAGGCTTG | 59 |
| 41 | Unigene11378_All | 5P | Zhang et al. 2017 | AgC11378F | GGAGCTGCAGAAAACCATTC | AgC11378R | GGTCCTGCTAGAAATGCAATG | 59 |
| 42 | Unigene1357_All | 5P | Zhang et al. 2017 | AgC1357F | GGGCTCTCCTCCTGGTTTAG | AgC1357R | GACACCAAATCGCTTCGTC | 59 |
| 43 | Unigene3925_All | 5P | Zhang et al. 2017 | AgC3925F | GTTGAACCTGGGTACAAATTGATC | AgC3925R | GTGTTCTTGTTGGGTTCCGA | 59 |
| 44 | Unigene8886_All | 5P | Zhang et al. 2017 | AgC8886F | TAGCTGGCTTCACATTGCTG | AgC8886R | AGTTGGATGGAAGGCACAAC | 59 |
| 45 | Unigene13331_All | 5P | Zhang et al. 2017 | AgC13331F | GTTGTTTCAGACACCGCAAG | AgC13331R | CAATTCATTCGTCCCCAATC | 59 |
| 46 | Unigene28341_All | 5P | Zhang et al. 2017 | AgC28341F | AACAAGCATCCCGACAAGAT | AgC28341R | CTTGTTGAAAGCACCACGAG | 59 |
| 47 | Unigene176_All | 5P | Zhang et al. 2017 | AgC176F | CTGATCATATGAATGCATAGCAGTG | AgC176R | TTGCAATCGAAAGGGTTGG | 59 |
| 48 | Unigene30401_All | 5P | Zhang et al. 2017 | AgC30401F | AAGTCGCTGGAGAGGAAGGT | AgC30401R | CTCCACATTGCTGATTGCAG | 59 |
| 49 | Unigene3513_All | 5P | Zhang et al. 2017 | AgC3513F | CTACCAAATGCCTGCCTGTT | AgC3513R | ATGAAGCAACGTCACACAGC | 59 |
| 50 | Unigene65139_All | 5P | Zhang et al. 2017 | AgC65139F | CCTGATGATCACGCTCATGC | AgC65139R | TGACATACAAAACATCAAATCTCGT | 59 |
| 51 | Unigene9517_All | 5P | Zhang et al. 2017 | AgC9517F | CGCTAATCAACCACTTGCAG | AgC9517R | ACCACACCACATCCCAATTT | 59 |
| 52 | Unigene9705_All | 5P | Zhang et al. 2017 | AgC9705F | TGATTGGCTTGGTTTTTATGC | AgC9705R | ACATTCAGCAGCGAGTGTTG | 59 |
| 53 | Unigene9736_All | 5P | Zhang et al. 2017 | AgC9736F | ATCCTGCAGCTGCATCCTCT | AgC9736R | GACCCAAATCGTCCTGATGAG | 59 |
| 54 | Unigene31661_All | 5P | Zhang et al. 2017 | AgC31661F | AATCACCGAGAAGGTCGATG | AgC31661R | CACTCTGCAAGCACCACATT | 59 |
| 55 | Unigene2896_All | 5P | Zhang et al. 2017 | AgC2896F | ATCTCTGTTCATGCCCCATC | AgC2896R | CTGTACAGGGCAAGGGTGAT | 59 |
| 56 | Unigene8391_All | 5P | Zhang et al. 2017 | AgC8391F | CCGTGTCTGTGACTCAGCAT | AgC8391R | GCACAAGACAGAGTCGGTGA | 59 |
| 57 | Unigene10666_All | 5P | Zhang et al. 2017 | AgC10666F | CACTGGCTATACCAGCACGA | AgC10666R | CGGTCAACAATGGAGGTTTT | 59 |
| 58 | Unigene13690_All | 5P | Zhang et al. 2017 | AgC13690F | GGCGAGAGTACGGTAACCTG | AgC13690R | TGAACGACATGGATGGTTTC | 59 |
| 59 | Unigene737_All | 5P | Zhang et al. 2017 | AgC737F | GCTTGCTGGATAGTAGCTCGAG | AgC737R | CTACTACTAGCACCATAACGAAACG | 59 |
| 60 | Unigene32771_All | 5P | Zhang et al. 2017 | AgC32771F | GAGCCGGTCCTAGCCTAACT | AgC32771R | CACAATATCTGAACCAGGCGTC | 59 |
| 61 | Unigene6591_All | 5P | Zhang et al. 2017 | AgC6591F | GAGGGGGCTTACTCCTTGAC | AgC6591R | CTATGCATAAAGGGCCTGGA | 59 |
| 62 | Unigene24589_All | 5P | Zhang et al. 2017 | AgC24589F | TGACGTGACATCATAACCCTTG | AgC24589R | GGAGGTCGACTAAACAGTCGGA | 59 |
| 63 | Unigene10460_All | 5P | Zhang et al. 2017 | AgC10460F | CCATGGATCTGGGTCAAGTT | AgC10460R | AGGACTGCCTCTCCACCAC | 59 |
| 64 | Unigene18817_All | 5P | Zhang et al. 2017 | AgC18817F | CCAATCCATTCAGCCACAGA | AgC18817R | CGTCATGAAAGTTGACCCCA | 59 |
| 65 | Unigene36915_All | 5P | Zhang et al. 2017 | AgC36915F | ACGGCATGGAGAACGGGTAG | AgC36915R | TAGGCTGCTGCAGGACCTGA | 59 |
| 66 | Unigene9729_All | 5P | Zhang et al. 2017 | AgC9729F | CGTGAACGTGATCGCTACAG | AgC9729R | CCACCAGTCTGTTTTGAGGA | 59 |
| 67 | Unigene21998_All | 5P | Zhang et al. 2017 | AgC21998F | AGAAGAGGAGCCGGGAGAG | AgC21998R | GCTGTCCACATCGACATGCT | 59 |
| 68 | Unigene5555_All | 5P | Zhang et al. 2017 | AgC5555F | AAAGGTTCTGCTGCTGCTGC | AgC5555R | AAACCACCCATGAAATGGAAC | 59 |
| 69 | Unigene23589_All | 5P | Zhang et al. 2017 | AgC23589F | CGGCTCCCCCGTAAAAGC | AgC23589R | GGACGGGTAGGTGCCTGAG | 59 |
| 70 | Unigene69236_All | 5P | Zhang et al. 2017 | AgC69236F | TCCTCTTTTCCAACTGAGCA | AgC69236R | TGGCAACTAACGACAGGAAG | 59 |
| 71 | Unigene7287_All | 5P | Zhang et al. 2017 | AgC7287F | CACTTTCTTTGGGAGCAAGC | AgC7287R | CCGAAGTCCATGTTTTTCCA | 59 |
| 72 | Unigene10646_All | 5P | Zhang et al. 2017 | AgC10646F | GGCTAAGCTTTTGAGAAGTGACA | AgC10646R | TTAGGACTCAGACCAGCAGTTTT | 59 |
| 73 | Unigene12168_All | 5P | Zhang et al. 2017 | AgC12168F | TATTGCGTTCGCAAGTTCAC | AgC12168R | CATCATGCAAATCCATGTCC | 59 |
| 74 | Unigene24483_All | 5P | Zhang et al. 2017 | AgC24483F | AACGCCAAGTACATCGAGGA | AgC24483R | CTTTCTCCCTCCACTCCGAT | 59 |
| 75 | Unigene9605_All | 5P | Zhang et al. 2017 | AgC9605F | CCTGCTTCCTGGTAGCTTTG | AgC9605R | GCCCAAACACCTCCAACG | 59 |
| 76 | Unigene63115_All | 5P | Zhang et al. 2017 | AgC63115F | GACTCCCGCTGCATCATC | AgC63115R | GTGTCGGGAGAGAGAGCTGA | 59 |
| 77 | Unigene1999_All | 5P | Zhang et al. 2017 | AgC1999F | GGTCTCTCGGGTATGCTTTG | AgC1999R | CATCTCAGGGGCCACTCTAC | 59 |
| 78 | Unigene68911_All | 5P | Zhang et al. 2017 | AgC68911F | GTTGCGGTTTTCGATTTGTT | AgC68911R | CACCTCTAGCTCATGCACCA | 59 |
| 79 | Unigene10067_All | 5P | Zhang et al. 2017 | AgC10067F | TGTGGGAAGCTAGATTGCAT | AgC10067R | CCGTATTATGCACGGTTGGT | 59 |
| 80 | Unigene30260_All | 5P | Zhang et al. 2017 | AgC30260F | ATCTGTCCAGCACATGCAAC | AgC30260R | ACCTCAAGGAGTGCAACTGG | 59 |
| 81 | Unigene50358_All | 5P | Zhang et al. 2017 | AgC50358F | AGGGTCAGGTTCTCCTCCTC | AgC50358R | CTCAGCCGCTTCGAGATG | 59 |
| 82 | Unigene21201_All | 5P | Zhang et al. 2017 | AgC21201F | TGTGATACCTACTTTGCCTGTTTT | AgC21201R | CATGGAAATCAAGGAAAATAATTG | 59 |
| 83 | Unigene1555_All | 5P | Zhang et al. 2017 | AgC1555F | TGCCACGGATCAAATGAGTA | AgC1555R | TGCACTCCCGTAAGTGAACA | 59 |
| 84 | Unigene1591_All | 5P | Zhang et al. 2017 | AgC1591F | GGGAACTACTCGCATAGGGC | AgC1591R | GAAACCATCCGTCGAACCTC | 59 |
| 85 | Unigene24378_All | 5P | Zhang et al. 2017 | AgC24378F | ATGGGGATGAGCCCATGA | AgC24378R | TTTCATCCCGAGCGCTGA | 59 |
| 86 | Unigene27081_All | 5P | Zhang et al. 2017 | AgC27081F | GTACGTGCTCGCTGCAAAT | AgC27081R | TGCTGTTGCTGTGCATTGTA | 59 |
| 87 | Unigene66523_All | 5P | Zhang et al. 2017 | AgC66523F | GGTTCAGAGGGTTGACCAAA | AgC66523R | ATGACGATTTCGGATCCTTG | 59 |
| 88 | Unigene13942_All | 5P | Zhang et al. 2017 | AgC13942F | GAGGCATTTGGACCAAGAGA | AgC13942R | TCGGGCGATCTCTACCATAG | 59 |
| 89 | Unigene71053_All | 5P | Zhang et al. 2017 | AgC71053F | GAACATTGCTTCTGGGGAAA | AgC71053R | AGGGTGTCATCCTCTTCGTG | 59 |
| 90 | Unigene23725_All | 5P | Zhang et al. 2017 | AgC23725F | GCCAGTTAGCCTTGCACCTG | AgC23725R | GGCCAGCACAATACTTCTATGC | 59 |
| 91 | Unigene28174_All | 5P | Zhang et al. 2017 | AgC28174F | TGGAGTTATAGACTCCTGGGATTAC | AgC28174R | TCTGCATCATAACTTTGGGTCC | 59 |
| 92 | Unigene47086_All | 5P | Zhang et al. 2017 | AgC47086F | CATTCTGTAGCATTGGCATGT | AgC47086R | CAGCTGGCTGTTGTTTTGTG | 59 |
| 93 | Unigene9641_All | 5P | Zhang et al. 2017 | AgC9641F | CGGGAGCGATGGATTACTGT | AgC9641R | CACCTCCTTACAAGCAGCTATTC | 59 |
| 94 | Unigene4724_All | 5P | Zhang et al. 2017 | AgC4724F | CCCAAGTAGATCATCCATCC | AgC4724R | TATTACTTAAATCACCTCAATTAGTG | 59 |
| 95 | Unigene24532_All | 5P | Zhang et al. 2017 | AgC24532F | CCATGGTACTTGTCAGGCGG | AgC24532R | TGGTCTCTCAGGGAAATGGC | 59 |
| 96 | Unigene27556_All | 5P | Zhang et al. 2017 | AgC27556F | CGTGCCGATCTTGAGCTTGT | AgC27556R | TTCGCTACAACTCGGCAGAGA | 59 |
| 97 | Unigene5693_All | 5P | Zhang et al. 2017 | AgC5693F | TCTCAAAAGGAAGGCCCCTA | AgC5693R | TGGTTCAACTGGTTTAACCGTC | 59 |
| 98 | Unigene4849_All | 5P | Zhang et al. 2017 | AgC4849F | CCTCAACTCCTTCTGCAACC | AgC4849R | CTTAACAAGTGCCAGCGTCA | 59 |
| 99 | Unigene7676_All | 5P | Zhang et al. 2017 | AgC7676F | TGGTGGCATATATCCCAGTGTT | AgC7676R | AATGGAGAAGGCGTTACCGA | 59 |
| 100 | Unigene9285_All | 5P | Zhang et al. 2017 | AgC9285F | GATCCAAAGAATGCCCTGTC | AgC9285R | TCCCAATTCTTTGTGCATTG | 59 |
| 101 | Unigene30264_All | 5P | Zhang et al. 2017 | AgC30264F | TCGGCATATTCTGCCCAAAC | AgC30264R | GAGATATGGGAAGGCAAGGATC | 59 |
| 102 | Unigene9438_All | 5P | Zhang et al. 2017 | AgC9438F | AATGTGATGTACGAAGGTCATCC | AgC9438R | CTGATTTAGTGCGCACTATGGA | 59 |
| 103 | Unigene23959_All | 5P | Zhang et al. 2017 | AgC23959F | CGATCAAAACACAAATATCCCAAG | AgC23959R | GCTAGCTTCTAGCTCATCAGATGC | 59 |
| 104 | Unigene7202_All | 5P | Zhang et al. 2017 | AgC7202F | TAGGAAGGAAGGTTGCATGG | AgC7202R | CTTTCCCTCTCGACTGATGC | 59 |
| 105 | Unigene6183_All | 5P | Zhang et al. 2017 | AgC6183F | CAACTCAAACGGTGAGGTGA | AgC6183R | CCAAAGCCAAGTCAAGAGGA | 59 |
| 106 | Unigene7482_All | 5P | Zhang et al. 2017 | AgC7482F | CCTCTGAGCGTTGCACTACA | AgC7482R | GACCCTCCCTCTGACATTGA | 59 |
| 107 | Unigene13641_All | 5P | Zhang et al. 2017 | AgC13641F | TGCACCATGGGAAATTATGA | AgC13641R | GCCACTTCTCTTCGCATTTC | 59 |
| 108 | Unigene9485_All | 5P | Zhang et al. 2017 | AgC9485F | GATGTCCACACACTCGATGC | AgC9485R | CATTCGCTGCACAAATTCTC | 59 |
| 109 | Unigene13130_All | 5P | Zhang et al. 2017 | AgC13130F | ACAGAGCTGGAGAAGCGAAG | AgC13130R | ATGGACAGAGGGAGGATGC | 59 |
| 110 | Unigene11679_All | 5P | Zhang et al. 2017 | AgC11679F | AATGTAGCCAACAACGTCCA | AgC11679R | CCTGTTTGTTCAGGCACTCA | 59 |
| 111 | Unigene68097_All | 5P | Zhang et al. 2017 | AgC68097F | GTTGTTTTCGGTGCCTGTCT | AgC68097R | CACACGAGGTGCAAAAGAAA | 59 |
| 112 | Unigene62402_All | 5P | Zhang et al. 2017 | AgC62402F | CCTCTGATCTCCCTGCTCTG | AgC62402R | ACGGCATTGCAGAAAAGCTA | 59 |
| 113 | Unigene9447_All | 5P | Zhang et al. 2017 | AgC9447F | TCACTCCCTTTACCTGTGTGTGTA | AgC9447R | CCTCCGTCAGCATTTACAGAAA | 59 |
| 114 | Unigene58028_All | 5P | Zhang et al. 2017 | AgC58028F | TCTGACATGAACAGCGGAAC | AgC58028R | GATCAAGGACCCATGCATTT | 59 |
| 115 | Unigene12001_All | 5P | Zhang et al. 2017 | AgC12001F | GAGAGAGGGAGCTGGCTTTT | AgC12001R | CCGCATCCTGTGGCTATTAC | 59 |
| 116 | Unigene1216_All | 5P | Zhang et al. 2017 | AgC1216F | TTGTGCGATAGCCATTCAAA | AgC1216R | ACGCTTGCTGGCTCACATA | 59 |
| 117 | Unigene30329_All | 5P | Zhang et al. 2017 | AgC30329F | TTGATTGGCTCAAAACATGG | AgC30329R | AGTCCAACGCAACCTCATCT | 59 |
| 118 | Unigene9548_All | 5P | Zhang et al. 2017 | AgC9548F | GCCCTAGCGATTCAGTCAAA | AgC9548R | ATGCGAGCTCCAATCTTTGT | 59 |
| 119 | Unigene10284_All | 5P | Zhang et al. 2017 | AgC10284F | CATCGCACCTCCCTGTTATC | AgC10284R | GTGGTTCCAGTAGCCAGCA | 59 |
| 120 | Unigene11525_All | 5P | Zhang et al. 2017 | AgC11525F | TGGTACGACGCAGTGTTTTC | AgC11525R | GGGGCAATCTTCACCAAACT | 59 |
| 121 | Unigene13145_All | 5P | Zhang et al. 2017 | AgC13145F | TCGAGACAACCCTAAGATGGA | AgC13145R | TGCAGTCACACGTGCAGTTA | 59 |
| 122 | Unigene24857_All | 5P | Zhang et al. 2017 | AgC24857F | GGTGCCCTTCCTTGGATACT | AgC24857R | CATGAGAGAACACCGGATCA | 59 |
| 123 | Unigene27729_All | 5P | Zhang et al. 2017 | AgC27729F | GTCGAAGTTGTCCAGCAGGT | AgC27729R | CTTGCCATTTCCTCCCTTCT | 59 |
| 124 | Unigene55208_All | 5P | Zhang et al. 2017 | AgC55208F | ATGTGTGAGCAAGTGTATGC | AgC55208R | CGTGCATTAACCATCTCTCT | 59 |
| 125 | Unigene1262_All | 5P | Zhang et al. 2017 | AgC1262F | CCTGATTGGCTGTCAACGAC | AgC1262R | GACTCACCATGTTGGGCTTT | 59 |
| 126 | Unigene9468_All | 5P | Zhang et al. 2017 | AgC9468F | TGCCAAACTTGTGGTCTGGT | AgC9468R | GTGACTTTGAGCCGATGGAA | 59 |
| 127 | Unigene14482_All | 5P | Zhang et al. 2017 | AgC14482F | GCAAAACTCCGAGACAATCC | AgC14482R | TGGCGGTAGTACAATCTTGG | 59 |
| 128 | Unigene16436_All | 5P | Zhang et al. 2017 | AgC16436F | CCTCGGGAAATCTGATGTAGTTC | AgC16436R | GCCTTGTCTTCGATCTGCCT | 59 |
| 129 | Unigene39546_All | 5P | Zhang et al. 2017 | AgC39546F | TGCAGTTTTCTGGTCCATTG | AgC39546R | CCTAGTGGGTGCCAAGTTGT | 59 |
| 130 | Unigene55913_All | 5P | Zhang et al. 2017 | AgC55913F | GCAACAGGCGTGGAGTTC | AgC55913R | CAAAACCGTTCGACTGATCA | 59 |
| 131 | Unigene62782_All | 5P | Zhang et al. 2017 | AgC62782F | GCCAACAAGCAGTGAGTCAA | AgC62782R | GAAGCATTTTCGAGGAGCAG | 59 |
| 132 | Unigene7997_All | 5P | Zhang et al. 2017 | AgC7997F | AAGATGGTGACGGGGTTGAC | AgC7997R | CATTGACGCCGTCTGACTTC | 59 |
| 133 | Unigene5865_All | 5P | Zhang et al. 2017 | AgC5865F | CGAGGATGAGAAGGATCAGCA | AgC5865R | CAAGCAGTGCCCAAGCTACT | 59 |
| 134 | Unigene21276_All | 5P | Zhang et al. 2017 | AgC21276F | TCGTCTTAGTCGGCCATCTC | AgC21276R | TTTTCCTACTCCGCCTGTTG | 59 |
| 135 | Unigene9456_All | 5P | Zhang et al. 2017 | AgC9456F | ATCAATTCACATCTCAGCACAGAC | AgC9456R | CCCTTCTGTTAGGGAGTCTGTTTA | 59 |
| 136 | Unigene34768_All | 5P | Zhang et al. 2017 | AgC34768F | CATGAGATCGGAAATCTCGTG | AgC34768R | CAGACAATTGGTTTCGGGATAT | 59 |
| 137 | Unigene59195_All | 5P | Zhang et al. 2017 | AgC59195F | GGATCTGGCGACAACAGG | AgC59195R | GAGGAGGAGGCGGAAGAC | 59 |
| 138 | Unigene26591_All | 5P | Zhang et al. 2017 | AgC26591F | GTACCGAATCGAATCCTGCT | AgC26591R | TGCCATGCGGAAGTACAATA | 59 |
| 139 | Unigene9095_All | 5P | Zhang et al. 2017 | AgC9095F | CTTTGCATTGGTCGCAGTTA | AgC9095R | AGCAGTCATCTGGAGGCAGT | 59 |
| 140 | Unigene979_All | 5P | Zhang et al. 2017 | AgC979F | ATCGATGGATCGGAACAAAA | AgC979R | CAACGAAGGACGCTGTTACG | 59 |
| 141 | Unigene12409_All | 5P | Zhang et al. 2017 | AgC12409F | GGTGCCTCTCTTTCCTCCTT | AgC12409R | TCCTCCTTTGGTTGTTCTCG | 59 |
| 142 | Unigene63662_All | 5P | Zhang et al. 2017 | AgC63662F | CCGGTCAGCGTTTTGACTAT | AgC63662R | GACGCTGACTGACGAAATCA | 59 |
| 143 | Unigene1882_All | 5P | Zhang et al. 2017 | AgC1882F | CTCTGTTGGCCCAAGTTTTC | AgC1882R | ATGCGAGGCAACAATGAAAT | 59 |
| 144 | Unigene23263_All | 5P | Zhang et al. 2017 | AgC23263F | GGCCGAGAAAGCTCACAATA | AgC23263R | TCTCAGCAGCCTCGTGTATG | 59 |
| 145 | Unigene9266_All | 5P | Zhang et al. 2017 | AgC9266F | GCATAGGTATGGACACGAGGA | AgC9266R | CATCGAGGCCGATCTTTATC | 59 |
| 146 | Unigene11072_All | 5P | Zhang et al. 2017 | AgC11072F | CAAAGCGCAGCTTGACTCTA | AgC11072R | CCCCAGCATACTCGAGAGAG | 59 |
| 147 | Unigene45687_All | 5P | Zhang et al. 2017 | AgC45687F | CGATCTGTCTGTCATCCGAAGT | AgC45687R | AACGGGTTGCCGTACTCGT | 59 |
| 148 | Unigene6571_All | 5P | Zhang et al. 2017 | AgC6571F | TGCACTTGAGCCTTGTCCTA | AgC6571R | CTCCAGCATTTGCCTTTCTC | 59 |
| 149 | Unigene9923_All | 5P | Zhang et al. 2017 | AgC9923F | TTCTCCTCCGGACGTACAAG | AgC9923R | TTGTCCGGTTGTTCAGCATA | 59 |
| 150 | Unigene9381_All | 5P | Zhang et al. 2017 | AgC9381F | CATCCAAGATCCAACCAACATT | AgC9381R | ATATCTGTCCGCCTGAACCG | 59 |
| 151 | Unigene10442_All | 5P | Zhang et al. 2017 | AgC10442F | TCCCACAGCCAATTTTCATT | AgC10442R | CACAGGACCAAGGCACTCTT | 59 |
| 152 | Unigene9342_All | 5P | Zhang et al. 2017 | AgC9342F | CATCCTCAAGGACTACACCACC | AgC9342R | ACTCAATTATTCACACCCCGATA | 59 |
| 153 | Unigene6771_All | 5P | Zhang et al. 2017 | AgC6771F | TGCGCAAAAGGAGATAGAGG | AgC6771R | ATTGTTTGGACGAGGCTACG | 59 |
| 154 | Unigene66575_All | 5P | Zhang et al. 2017 | AgC66575F | TCGGATTCCTCCTATCAACG | AgC66575R | AAGTGTTAGGCTCTGGCGAA | 59 |
| 155 | Unigene30595_All | 5P | Zhang et al. 2017 | AgC30595F | GCAAGGTGGCTTTTCATGTC | AgC30595R | CTTACGAGGCATTCATGTACAGTTT | 59 |
| 156 | Unigene1839_All | 5P | Zhang et al. 2017 | AgC1839F | GACATCTACAACCTCCTGCTGG | AgC1839R | GTTCGAGACTTGATAAACGGTGA | 59 |
| 157 | Unigene22316_All | 5P | Zhang et al. 2017 | AgC22316F | CAGGGACTGGATGCCTCTCT | AgC22316R | CGCCATCCTCCATCATCTTCTT | 59 |
| 158 | Unigene22382_All | 5P | Zhang et al. 2017 | AgC22382F | CATGGCCGTTCTGCTTCCT | AgC22382R | CACTTGCTTTGGTTCCTCTCG | 59 |
| 159 | Unigene7941_All | 5P | Zhang et al. 2017 | AgC7941F | GGTGGGAGATTTAGTTCTGGATC | AgC7941R | ATCACCCTCGGGCTCAAAC | 59 |
| 160 | Unigene9457_All | 5P | Zhang et al. 2017 | AgC9457F | GGCTATTTTGCCAAACTGGA | AgC9457R | AGTGGAGGGTAATGCAAGACAA | 59 |
| 161 | Unigene10086_All | 2P | Zhang et al. 2017 | AgC10086F | GTTGAGGCATGCCGTCTTAG | AgC10086R | CCTAGGCGCAGCTAATTCAC | 59 |
| 162 | Unigene12165_All | 2P | Zhang et al. 2017 | AgC12165F | CGTTCACCTCTCCCTGTTGT | AgC12165R | GTCATCGTGCATCGCCTACT | 59 |
| 163 | Unigene368_All | 2P | Zhang et al. 2017 | AgC368F | TTTGTAGGAGTAATTGTTGTCAAGC | AgC368R | TGCCAATCCCTGTAAGATGC | 59 |
| 164 | Unigene10757_All | 2P | Zhang et al. 2017 | AgC10757F | AAGAGGGACCTGCCGAAG | AgC10757R | CTCAGATTTTGCGGAGCAC | 59 |
| 165 | Unigene924_All | 2P | Zhang et al. 2017 | AgC924F | CTTGCACAAAAGAGCACCAA | AgC924R | CCTCTTGGCAGGCATATGTT | 59 |
| 166 | Unigene31586_All | 2P | Zhang et al. 2017 | AgC31586F | GCACAAATGGAGAGCACAGA | AgC31586R | AAGCAAGCTCACTCGCACTT | 59 |
| 167 | Unigene353_All | 2P | Zhang et al. 2017 | AgC353F | AGAGGCCGTCGTCTCCCA | AgC353R | TGCAACAACACGCAGCAGC | 59 |
| 168 | Unigene3425_All | 2P | Zhang et al. 2017 | AgC3425F | CAGGGGATCAGTACCCAAGC | AgC3425R | TCATGTGGTCATTGGTTGCA | 59 |
| 169 | Unigene10935_All | 2P | Zhang et al. 2017 | AgC10935F | CTGAAAGCAACACATCTTCTGTG | AgC10935R | TACACGCTGACAGTCTTAAACGA | 59 |
| 170 | Unigene24824_All | 2P | Zhang et al. 2017 | AgC24824F | ATACACCTGCAATGGCATTTG | AgC24824R | TGACCCGATTCCATCTCTGAG | 59 |
| 171 | Unigene19918_All | 2P | Zhang et al. 2017 | AgC19918F | GTCAAAAACAAGTGCGGTGT | AgC19918R | CTGGAATAACCATCACGAAAT | 59 |
| 172 | Unigene26300_All | 2P | Zhang et al. 2017 | AgC26300F | GGTCGGTAGGGGGTCTGTAT | AgC26300R | GGTCAACTGCCTGTGAAACA | 59 |
| 173 | Unigene788_All | 2P | Li et al. 2016 | AgC788F | ACAATTGGGCTTTGTGAAGG | AgC788R | CGACGGTAAAAATTGGATTG | 59 |
| 174 | Unigene32132_All | 2P | Li et al. 2016 | AgC32132F | CCACGGGATTAAGGAGGAGT | AgC32132R | CCCAGCAGGTCTGCATCTAT | 59 |
| 175 | Unigene31650_All | 2P | Li et al. 2016 | AgC31650F | GTTCAGTTGTGAGGCGGTCT | AgC31650R | GCTCTGCTGCTGCACCTT | 59 |
| 176 | Unigene1846_All | 2P | Li et al. 2016 | AgC1846F | ATGCATTTCTCCTGCCAGAC | AgC1846R | GGACACTGGTGTTGATGTGC | 59 |
| 177 | Unigene4988_All | 2P | Li et al. 2016 | AgC4988F | CGATCAAAACGGGAAATTGT | AgC4988R | TTGGCCTCTGTAGCAAGTCA | 59 |
| 178 | Unigene10705_All | 2P | Li et al. 2016 | AgC10705F | TCCCTAATCCAGCCCCTATC | AgC10705R | GCACAACTAGCTCCCCATGT | 59 |
| 179 | Unigene23139_All | 2P | Li et al. 2016 | AgC23139F | GGCCCCTTTAGATCTCTACCA | AgC23139R | TGTTCAGACATTTGAAGGATGTG | 59 |
| 180 | Unigene25535_All | 2P | Li et al. 2016 | AgC25535F | GGATCGGATCGTCCTTAGGT | AgC25535R | CGGCTACCTTTTGGTTCCTT | 59 |
| 181 | Unigene27067_All | 2P | Li et al. 2016 | AgC27067F | ATGGTTGGGATCCTTGACTTG | AgC27067R | TCAAGCGTAATCTCAACTTGACC | 59 |
| 182 | Unigene31410_All | 2P | Li et al. 2016 | AgC31410F | GCGACCGTATCCATGGTATG | AgC31410R | TGTGACATTTCCATCATCTGGA | 59 |
| 183 | Unigene50604_All | 2P | Li et al. 2016 | AgC50604F | TGGCATTTTGCATTTGACTG | AgC50604R | TGCAACTAGCTCCACCCTTT | 59 |
| 184 | Unigene1609_All | 2P | Li et al. 2016 | AgC1609F | AATAACCATTTCCCGCACAG | AgC1609R | CGGAGGGAGTAGAATGCAAG | 59 |
| 185 | Unigene25348_All | 2P | Li et al. 2016 | AgC25348F | CAGGCACATACCTCACAGTGC | AgC25348R | TACAACTTCGGCCAAATTCG | 59 |
| 186 | Unigene26072_All | 2P | Li et al. 2016 | AgC26072F | ATGTAACATGACGCCGATGA | AgC26072R | ACAAACTGCACCCGACAAG | 59 |
| 187 | Unigene27068_All | 2P | Li et al. 2016 | AgC27068F | CTTCCGTTCTCGTGATGGTT | AgC27068R | CAGGTCCAAATGTCCAATCT | 59 |
| 188 | Unigene27928_All | 2P | Li et al. 2016 | AgC27928F | ATTGGGAGGCTACGGAAGAG | AgC27928R | AAATGCTCCAACAGGACGAG | 59 |
| 189 | Unigene33022_All | 2P | Li et al. 2016 | AgC33022F | GGTGGCACGACAGAAGAAGT | AgC33022R | GCACGACTGGATTGGCTATT | 59 |
| 190 | Unigene50203_All | 2P | Li et al. 2016 | AgC50203F | CACAGAGATCACCCTTCGTG | AgC50203R | GCAGCTGTAGGGGCATGT | 59 |
| 191 | Unigene50258_All | 2P | Li et al. 2016 | AgC50258F | GGGGTCCTGGTGAAAATAATG | AgC50258R | GCCGGAATCAATTTTGAGTAAC | 59 |
| 192 | Unigene51218_All | 2P | Li et al. 2016 | AgC51218F | GCTTGATTAAGCTCTGCTAAAGG | AgC51218R | ACCGATGATTCACGAGGAAC | 59 |
| 193 | Unigene54461_All | 2P | Li et al. 2016 | AgC54461F | ATATGATGGCCTTTGGCTCA | AgC54461R | GGATTCATGCAAGTCAGCAG | 59 |
| 194 | Unigene54558_All | 2P | Li et al. 2016 | AgC54558F | TGCAGCCTACCTGATCAAATC | AgC54558R | TGACATTGCGAAACAATGAAG | 59 |
| 195 | Unigene54388_All | 2P | Li et al. 2016 | AgC54388F | ATGCCCTACAGTCCTGCAAC | AgC54388R | ATTTCACAGAAGCCGCTACG | 59 |
| 196 | Unigene29835_All | 2P | Li et al. 2016 | AgC29835F | ATTATGCGTTCAGCCTTGTG | AgC29835R | CGCGATATCAAATCCCTCAT | 59 |
| 197 | Unigene31300_All | 2P | Li et al. 2016 | AgC31300F | AGTGTGGTTTGGGGTGATGT | AgC31300R | GCAAATGATTCCCAGGAAGA | 59 |
| 198 | Unigene10498_All | 2P | Li et al. 2016 | AgC10498F | CAAGGAGGCAGAAGCAATTC | AgC10498R | CCTCCCATCTCTTTAGCGAGT | 59 |
| 199 | Unigene32533_All | 2P | Li et al. 2016 | AgC32533F | GCCAAAGATGGTAGCCCTGTT | AgC32533R | AGCCTCCTTTACGAAACGGA | 59 |
| 200 | Unigene14191_All | 2P | Li et al. 2016 | AgC14191F | ACAACGCCTCTGCCACTACT | AgC14191R | TGCTCCATCGATCAATACCA | 59 |
| 201 | Unigene52107_All | 2P | Li et al. 2016 | AgC52107F | TCTTCCCCGACATCTCTCAC | AgC52107R | CCGAAGGTAGTGGCGGTA | 59 |
| 202 | Unigene10944_All | 2P | Li et al. 2016 | AgC10944F | GGGTCTTGGATGTACAGAAGC | AgC10944R | TTCGTGGAACATCAAGATGAA | 59 |
| 203 | Unigene19911_All | 2P | Li et al. 2016 | AgC19911F | GGGTGGTCATGAAAGGATTG | AgC19911R | TGCCGTAGCTTGTTCATCAG | 59 |
| 204 | Unigene29547_All | 2P | Li et al. 2016 | AgC29547F | ATGGTAAGCTCATGACATGCAG | AgC29547R | AGCCCCCATAGCTCTCTCAG | 59 |
| 205 | Unigene31114_All | 2P | Li et al. 2016 | AgC31114F | GACGGCATGTAATGCTGCTA | AgC31114R | AGTCGTGGAGCATAGCTTGG | 59 |
| 206 | Unigene3398_All | 2P | Li et al. 2016 | AgC3398F | AGAAACGCCAGAGGATCTGA | AgC3398R | GAAGCTACCTGAGGCTGCAC | 59 |
| 207 | Unigene4298_All | 2P | Li et al. 2016 | AgC4298F | TTGCTGAGGTCCAATTCCTC | AgC4298R | ACCTCCCGCTTAACCAATTT | 59 |
| 208 | Unigene17451_All | 2P | Li et al. 2016 | AgC17451F | ATGATGTCGCCTGAATCTCC | AgC17451R | ACACACCCCACAAAGAAAGC | 59 |
| 209 | Unigene33175_All | 2P | Li et al. 2016 | AgC33175F | GAGCCTTTCCTCTCCTCCAT | AgC33175R | CACATGGGAGTGCTCAGAAG | 59 |
| 210 | Unigene11994_All | 2P | Li et al. 2016 | AgC11994F | GGTCCATGACCATCATCCTC | AgC11994R | CCCAGGTGGAGACTCTTGAT | 59 |
| 211 | Unigene27581_All | 2P | Li et al. 2016 | AgC27581F | ATAGACAAACGGGACGGATTC | AgC27581R | ACAGATCCTTATCAGGGTTGTATTC | 59 |
| 212 | Unigene32544_All | 2P | Li et al. 2016 | AgC32544F | TTCGTCTTCGTCGGCAGACT | AgC32544R | TGTCGCTGATCTCTCCAACG | 59 |
| 213 | Unigene1560_All | 2P | Li et al. 2016 | AgC1560F | TTCACATCGCCATACGACAT | AgC1560R | AAGGAGCTCATTCAGCAAGC | 59 |
| 214 | Unigene12244_All | 2P | Li et al. 2016 | AgC12244F | TCAAACTGAAGCTCGGTATCC | AgC12244R | ACACATGCCATGGGAACTAA | 59 |
| 215 | Unigene14847_All | 2P | Li et al. 2016 | AgC14847F | CCAAGGCATCAAGGTTTTGT | AgC14847R | GCTACTCGGATGCAGACCTC | 59 |
| 216 | Unigene32182_All | 2P | Li et al. 2016 | AgC32182F | TGAAAGCTCGTGCCTTTGAT | AgC32182R | ATCGACCTGCTGCTCTTGTC | 59 |
| 217 | Unigene4115_All | 2P | Li et al. 2016 | AgC4115F | ACTCACGGTGCATGGTATGA | AgC4115R | TTGTGCTGTGCGTGTGTAAA | 59 |
| 218 | Unigene28398_All | 2P | Li et al. 2016 | AgC28398F | AAGCTGCTGCAAGATGACGT | AgC28398R | CTTGATGTTCTTCAGCCTCACAG | 59 |
| 219 | Unigene2847_All | 2P | Li et al. 2016 | AgC2847F | AGCATGAGGAATGGATCTGG | AgC2847R | CGGTGAAGCTCTGCAATATG | 59 |
| 220 | Unigene628_All | 2P | Li et al. 2016 | AgC628F | TGCAGTTTCAGTTTATTTCCTCTG | AgC628R | TTGAGTTTTCGATGCCTTCA | 59 |
| 221 | Unigene31991_All | 2P | Li et al. 2016 | AgC31991F | GATGCAGGCAGTTTTCCATC | AgC31991R | AGCACAGCTGTCCCACAATA | 59 |
| 222 | Unigene1439_All | 2P | Li et al. 2016 | AgC1439F | TCTTGTATCCAGCGTCATCG | AgC1439R | GGTTAATGCAAGGGATGTGG | 59 |
| 223 | Unigene5863_All | 2P | Li et al. 2016 | AgC5863F | TGCCGCTGAAAATTAAATCC | AgC5863R | TTTCTACAGCGATTCGTCCA | 59 |
| 224 | Unigene24233_All | 2P | Li et al. 2016 | AgC24233F | TCGATTTAGGCTGTGTCACG | AgC24233R | TCACCTTTGTGTATGTGTATTTCG | 59 |
| 225 | Unigene50281_All | 2P | Li et al. 2016 | AgC50281F | CCAATCAAATCCTTGTTCACC | AgC50281R | ACATGAGCCCGATGATTGTT | 59 |
| 226 | Unigene24649_All | 2P | Li et al. 2016 | AgC24649F | ATGGCCATCTCTCCATGC | AgC24649R | AAATCCCTCGACTGTTTAGGC | 59 |
| 227 | Unigene2289_All | 2P | Li et al. 2016 | AgC2289F | AAAAGCTAGGCGGTGGAAAG | AgC2289R | ATCCTGGTCCTGTGAGCAAC | 59 |
| 228 | Unigene53758_All | 2P | Li et al. 2016 | AgC53758F | AACAAACCATGCGCTGAAAC | AgC53758R | TTTGAACCAGCTCAGGAACC | 59 |
| 229 | Unigene13874_All | 2P | Li et al. 2016 | AgC13874F | GGAGCAGATCCAGTGCTACC | AgC13874R | AGACATCCACGAGGACAACC | 59 |
| 230 | Unigene2658_All | 2P | Li et al. 2016 | AgC2658F | CAGGCTTTTCCCCTATTACCA | AgC2658R | GGACCTTTAAACTGCAACCAA | 59 |
| 231 | Unigene27315_All | 2P | Li et al. 2016 | AgC27315F | TTTTTCCTGGGAGGTGTTGA | AgC27315R | GTGTGGCTAATGACGTGTGC | 59 |
| 232 | Unigene23021_All | 2P | Li et al. 2016 | AgC23021F | CAACTCGGTGCTGGTCGATG | AgC23021R | CTTGCAGATGAACATCACGCT | 59 |
| 233 | Unigene3725_All | 2P | Li et al. 2016 | AgC3725F | TGAGCAGAGACTTGGACTGG | AgC3725R | TTCGTTGTGGCTTCAAAGTG | 59 |
| 234 | Unigene1795_All | 2P | Li et al. 2016 | AgC1795F | CCTATCTTCGGTGACGCTGT | AgC1795R | AAAGCACAAACACCCTCCTG | 59 |
| 235 | Unigene3596_All | 2P | Li et al. 2016 | AgC3596F | ACAAATGCACATCGGTCAAA | AgC3596R | TGTCAACTCACGGCTGATTC | 59 |
| 236 | Unigene11777_All | 2P | Li et al. 2016 | AgC11777F | AGATCCAGGTGGTCTTTGGA | AgC11777R | GCAGCATCAGTTCCCACTTT | 59 |

Table S2 One hundred and fourteen EST-STS markers were detected in different wheat-*Agropyron cristatum* addition lines

| No. | **Unigene ID** | **EST-STS markers** | **Homoeologous  group** | ***A. cristatum*** Z559 | Fukuhokomugi | Ⅱ-3-1（1P） | Ⅱ-9-3（2P） | 736（3P） | Ⅱ-21-2（4P） | Ⅱ-11-1b（5P） | 4844-12（6P） | Ⅱ-5-1（7P） | Ⅱ-11-1（2P+5P） |
| --- | --- | --- | --- | --- | --- | --- | --- | --- | --- | --- | --- | --- | --- |
| 1 | Unigene9023_All | Agc9023 | 5P | ＋ | － | － | － | － | － | ＋ | － | － | ＋ |
| 2 | Unigene10395_All | Agc10395 | 5P | ＋ | － | － | － | － | － | ＋ | － | － | ＋ |
| 3 | Unigene10097_All | Agc10097 | 5P | ＋ | － | － | － | － | － | ＋ | － | － | ＋ |
| 4 | Unigene21866_All | Agc21866 | 5P | ＋ | － | － | － | － | － | ＋ | － | － | ＋ |
| 5 | Unigene49470_All | Agc49470 | 5P | ＋ | － | － | － | － | － | ＋ | － | － | ＋ |
| 6 | Unigene2175_All | Agc2175 | 5P | ＋ | － | － | － | － | － | ＋ | － | － | ＋ |
| 7 | Unigene32810_All | Agc32810 | 5P | ＋ | － | － | － | － | － | ＋ | － | － | ＋ |
| 8 | Unigene34035_All | Agc34035 | 5P | ＋ | － | － | － | － | － | ＋ | － | － | ＋ |
| 9 | Unigene4598_All | Agc4598 | 5P | ＋ | － | － | － | － | － | ＋ | － | － | ＋ |
| 10 | Unigene71979_All | Agc71979 | 5P | ＋ | － | － | － | － | － | ＋ | － | － | ＋ |
| 11 | Unigene32606_All | Agc32606 | 5P | ＋ | － | － | － | － | － | ＋ | － | － | ＋ |
| 12 | Unigene26558_All | Agc26558 | 5P | ＋ | － | － | － | － | － | ＋ | － | － | ＋ |
| 13 | Unigene9932_All | Agc9932 | 5P | ＋ | － | － | － | － | － | ＋ | － | － | ＋ |
| 14 | Unigene27139_All | Agc27139 | 5P | ＋ | － | － | － | － | － | ＋ | － | － | ＋ |
| 15 | Unigene13319_All | Agc13319 | 5P | ＋ | － | － | － | － | － | ＋ | － | － | ＋ |
| 16 | Unigene4457_All | Agc4457 | 5P | ＋ | － | － | － | － | － | ＋ | － | － | ＋ |
| 17 | Unigene72356_All | Agc72356 | 5P | ＋ | － | － | － | － | － | ＋ | － | － | ＋ |
| 18 | Unigene25041_All | Agc25041 | 5P | ＋ | － | － | － | － | － | ＋ | － | － | ＋ |
| 19 | Unigene22373_All | Agc22373 | 5P | ＋ | － | － | － | － | － | ＋ | － | － | ＋ |
| 20 | Unigene5771_All | Agc5771 | 5P | ＋ | － | － | － | － | － | ＋ | － | － | ＋ |
| 21 | Unigene22557_All | Agc22557 | 5P | ＋ | － | － | － | － | － | ＋ | － | － | ＋ |
| 22 | Unigene29276_All | Agc29276 | 5P | ＋ | － | － | － | － | － | ＋ | － | － | ＋ |
| 23 | Unigene35699_All | Agc35699 | 5P | ＋ | － | － | － | － | － | ＋ | － | － | ＋ |
| 24 | Unigene9229_All | Agc9229 | 5P | ＋ | － | － | － | － | － | ＋ | － | － | ＋ |
| 25 | Unigene4764_All | Agc4764 | 5P | ＋ | － | － | － | － | － | ＋ | － | － | ＋ |
| 26 | Unigene5477_All | Agc5477 | 5P | ＋ | － | － | － | － | － | ＋ | － | － | ＋ |
| 27 | Unigene3925_All | Agc3925 | 5P | ＋ | － | － | － | － | － | ＋ | － | － | ＋ |
| 28 | Unigene28341_All | Agc28341 | 5P | ＋ | － | － | － | － | － | ＋ | － | － | ＋ |
| 29 | Unigene30401_All | Agc30401 | 5P | ＋ | － | － | － | － | － | ＋ | － | － | ＋ |
| 30 | Unigene3513_All | Agc3513 | 5P | ＋ | － | － | － | － | － | ＋ | － | － | ＋ |
| 31 | Unigene9705_All | Agc9705 | 5P | ＋ | － | － | － | － | － | ＋ | － | － | ＋ |
| 32 | Unigene9736_All | Agc9736 | 5P | ＋ | － | － | － | － | － | ＋ | － | － | ＋ |
| 33 | Unigene737_All | Agc737 | 5P | ＋ | － | － | － | － | － | ＋ | － | － | ＋ |
| 34 | Unigene32771_All | Agc32771 | 5P | ＋ | － | － | － | － | － | ＋ | － | － | ＋ |
| 35 | Unigene10460_All | Agc10460 | 5P | ＋ | － | － | － | － | － | ＋ | － | － | ＋ |
| 36 | Unigene36915_All | Agc36915 | 5P | ＋ | － | － | － | － | － | ＋ | － | － | ＋ |
| 37 | Unigene9729_All | Agc9729 | 5P | ＋ | － | － | － | － | － | ＋ | － | － | ＋ |
| 38 | Unigene7287_All | Agc7287 | 5P | ＋ | － | － | － | － | － | ＋ | － | － | ＋ |
| 39 | Unigene12168_All | Agc12168 | 5P | ＋ | － | － | － | － | － | ＋ | － | － | ＋ |
| 40 | Unigene63115_All | Agc63115 | 5P | ＋ | － | － | － | － | － | ＋ | － | － | ＋ |
| 41 | Unigene30260_All | Agc30260 | 5P | ＋ | － | － | － | － | － | ＋ | － | － | ＋ |
| 42 | Unigene50358_All | Agc50358 | 5P | ＋ | － | － | － | － | － | ＋ | － | － | ＋ |
| 43 | Unigene1591_All | Agc1591 | 5P | ＋ | － | － | － | － | － | ＋ | － | － | ＋ |
| 44 | Unigene13942_All | Agc13942 | 5P | ＋ | － | － | － | － | － | ＋ | － | － | ＋ |
| 45 | Unigene23725_All | Agc23725 | 5P | ＋ | － | － | － | － | － | ＋ | － | － | ＋ |
| 46 | Unigene4724_All | Agc4724 | 5P | ＋ | － | － | － | － | － | ＋ | － | － | ＋ |
| 47 | Unigene24532_All | Agc24532 | 5P | ＋ | － | － | － | － | － | ＋ | － | － | ＋ |
| 48 | Unigene27556_All | Agc27556 | 5P | ＋ | － | － | － | － | － | ＋ | － | － | ＋ |
| 49 | Unigene5693_All | Agc5693 | 5P | ＋ | － | － | － | － | － | ＋ | － | － | ＋ |
| 50 | Unigene30264_All | Agc30264 | 5P | ＋ | － | － | － | － | － | ＋ | － | － | ＋ |
| 51 | Unigene7202_All | Agc7202 | 5P | ＋ | － | － | － | － | － | ＋ | － | － | ＋ |
| 52 | Unigene7482_All | Agc7482 | 5P | ＋ | － | － | － | － | － | ＋ | － | － | ＋ |
| 53 | Unigene11679_All | Agc11679 | 5P | ＋ | － | － | － | － | － | ＋ | － | － | ＋ |
| 54 | Unigene9447_All | Agc9447 | 5P | ＋ | － | － | － | － | － | ＋ | － | － | ＋ |
| 55 | Unigene1216_All | Agc1216 | 5P | ＋ | － | － | － | － | － | ＋ | － | － | ＋ |
| 56 | Unigene9548_All | Agc9548 | 5P | ＋ | － | － | － | － | － | ＋ | － | － | ＋ |
| 57 | Unigene11525_All | Agc11525 | 5P | ＋ | － | － | － | － | － | ＋ | － | － | ＋ |
| 58 | Unigene13145_All | Agc13145 | 5P | ＋ | － | － | － | － | － | ＋ | － | － | ＋ |
| 59 | Unigene27729_All | Agc27729 | 5P | ＋ | － | － | － | － | － | ＋ | － | － | ＋ |
| 60 | Unigene55208_All | Agc55208 | 5P | ＋ | － | － | － | － | － | ＋ | － | － | ＋ |
| 61 | Unigene9468_All | Agc9468 | 5P | ＋ | － | － | － | － | － | ＋ | － | － | ＋ |
| 62 | Unigene16436_All | Agc16436 | 5P | ＋ | － | － | － | － | － | ＋ | － | － | ＋ |
| 63 | Unigene39546_All | Agc39546 | 5P | ＋ | － | － | － | － | － | ＋ | － | － | ＋ |
| 64 | Unigene7997_All | Agc7997 | 5P | ＋ | － | － | － | － | － | ＋ | － | － | ＋ |
| 65 | Unigene21276_All | Agc21276 | 5P | ＋ | － | － | － | － | － | ＋ | － | － | ＋ |
| 66 | Unigene9456_All | Agc9456 | 5P | ＋ | － | － | － | － | － | ＋ | － | － | ＋ |
| 67 | Unigene9095_All | Agc9095 | 5P | ＋ | － | － | － | － | － | ＋ | － | － | ＋ |
| 68 | Unigene979_All | Agc979 | 5P | ＋ | － | － | － | － | － | ＋ | － | － | ＋ |
| 69 | Unigene23263_All | Agc23263 | 5P | ＋ | － | － | － | － | － | ＋ | － | － | ＋ |
| 70 | Unigene9266_All | Agc9266 | 5P | ＋ | － | － | － | － | － | ＋ | － | － | ＋ |
| 71 | Unigene45687_All | Agc45687 | 5P | ＋ | － | － | － | － | － | ＋ | － | － | ＋ |
| 72 | Unigene6571_All | Agc6571 | 5P | ＋ | － | － | － | － | － | ＋ | － | － | ＋ |
| 73 | Unigene9923_All | Agc9923 | 5P | ＋ | － | － | － | － | － | ＋ | － | － | ＋ |
| 74 | Unigene9342_All | Agc9342 | 5P | ＋ | － | － | － | － | － | ＋ | － | － | ＋ |
| 75 | Unigene6771_All | Agc6771 | 5P | ＋ | － | － | － | － | － | ＋ | － | － | ＋ |
| 76 | Unigene66575_All | Agc66575 | 5P | ＋ | － | － | － | － | － | ＋ | － | － | ＋ |
| 77 | Unigene22316_All | Agc22316 | 5P | ＋ | － | － | － | － | － | ＋ | － | － | ＋ |
| 78 | Unigene9457_All | Agc9457 | 5P | ＋ | － | － | － | － | － | ＋ | － | － | ＋ |
| 79 | Unigene10757_All | Agc10757 | 2P | ＋ | － | － | ＋ | － | － | － | － | － | ＋ |
| 80 | Unigene924_All | Agc924 | 2P | ＋ | － | － | ＋ | － | － | － | － | － | ＋ |
| 81 | Unigene10935_All | Agc10935 | 2P | ＋ | － | － | ＋ | － | － | － | － | － | ＋ |
| 82 | Unigene19918_All | Agc19918 | 2P | ＋ | － | － | ＋ | － | － | － | － | － | ＋ |
| 83 | Unigene788_All | Agc788 | 2P | ＋ | － | － | ＋ | － | － | － | － | － | ＋ |
| 84 | Unigene32132_All | Agc32132 | 2P | ＋ | － | － | ＋ | － | － | － | － | － | ＋ |
| 85 | Unigene31650_All | Agc31650 | 2P | ＋ | － | － | ＋ | － | － | － | － | － | ＋ |
| 86 | Unigene1846_All | Agc1846 | 2P | ＋ | － | － | ＋ | － | － | － | － | － | ＋ |
| 87 | Unigene10705_All | Agc10705 | 2P | ＋ | － | － | ＋ | － | － | － | － | － | ＋ |
| 88 | Unigene23139_All | Agc23139 | 2P | ＋ | － | － | ＋ | － | － | － | － | － | ＋ |
| 89 | Unigene31410_All | Agc31410 | 2P | ＋ | － | － | ＋ | － | － | － | － | － | ＋ |
| 90 | Unigene50604_All | Agc50604 | 2P | ＋ | － | － | ＋ | － | － | － | － | － | ＋ |
| 91 | Unigene25348_All | Agc25348 | 2P | ＋ | － | － | ＋ | － | － | － | － | － | ＋ |
| 92 | Unigene26072_All | Agc26072 | 2P | ＋ | － | － | ＋ | － | － | － | － | － | ＋ |
| 93 | Unigene51218_All | Agc51218 | 2P | ＋ | － | － | ＋ | － | － | － | － | － | ＋ |
| 94 | Unigene31300_All | Agc31300 | 2P | ＋ | － | － | ＋ | － | － | － | － | － | ＋ |
| 95 | Unigene32533_All | Agc32533 | 2P | ＋ | － | － | ＋ | － | － | － | － | － | ＋ |
| 96 | Unigene10944_All | Agc10944 | 2P | ＋ | － | － | ＋ | － | － | － | － | － | ＋ |
| 97 | Unigene19911_All | Agc19911 | 2P | ＋ | － | － | ＋ | － | － | － | － | － | ＋ |
| 98 | Unigene3398_All | Agc3398 | 2P | ＋ | － | － | ＋ | － | － | － | － | － | ＋ |
| 99 | Unigene4298_All | Agc4298 | 2P | ＋ | － | － | ＋ | － | － | － | － | － | ＋ |
| 100 | Unigene17451_All | Agc17451 | 2P | ＋ | － | － | ＋ | － | － | － | － | － | ＋ |
| 101 | Unigene11994_All | Agc11994 | 2P | ＋ | － | － | ＋ | － | － | － | － | － | ＋ |
| 102 | Unigene27581_All | Agc27581 | 2P | ＋ | － | － | ＋ | － | － | － | － | － | ＋ |
| 103 | Unigene1560_All | Agc1560 | 2P | ＋ | － | － | ＋ | － | － | － | － | － | ＋ |
| 104 | Unigene32182_All | Agc32182 | 2P | ＋ | － | － | ＋ | － | － | － | － | － | ＋ |
| 105 | Unigene4115_All | Agc4115 | 2P | ＋ | － | － | ＋ | － | － | － | － | － | ＋ |
| 106 | Unigene2847_All | Agc2847 | 2P | ＋ | － | － | ＋ | － | － | － | － | － | ＋ |
| 107 | Unigene31991_All | Agc31991 | 2P | ＋ | － | － | ＋ | － | － | － | － | － | ＋ |
| 108 | Unigene24233_All | Agc24233 | 2P | ＋ | － | － | ＋ | － | － | － | － | － | ＋ |
| 109 | Unigene50281_All | Agc50281 | 2P | ＋ | － | － | ＋ | － | － | － | － | － | ＋ |
| 110 | Unigene24649_All | Agc24649 | 2P | ＋ | － | － | ＋ | － | － | － | － | － | ＋ |
| 111 | Unigene2658_All | Agc2658 | 2P | ＋ | － | － | ＋ | － | － | － | － | － | ＋ |
| 112 | Unigene27315_All | Agc27315 | 2P | ＋ | － | － | ＋ | － | － | － | － | － | ＋ |
| 113 | Unigene1795_All | Agc1795 | 2P | ＋ | － | － | ＋ | － | － | － | － | － | ＋ |
| 114 | Unigene3596_All | Agc3596 | 2P | ＋ | － | － | ＋ | － | － | － | － | － | ＋ |

the presence of marker: +; the absence of marker: -

Figure S1 FISH identification of Ⅱ-11-1b


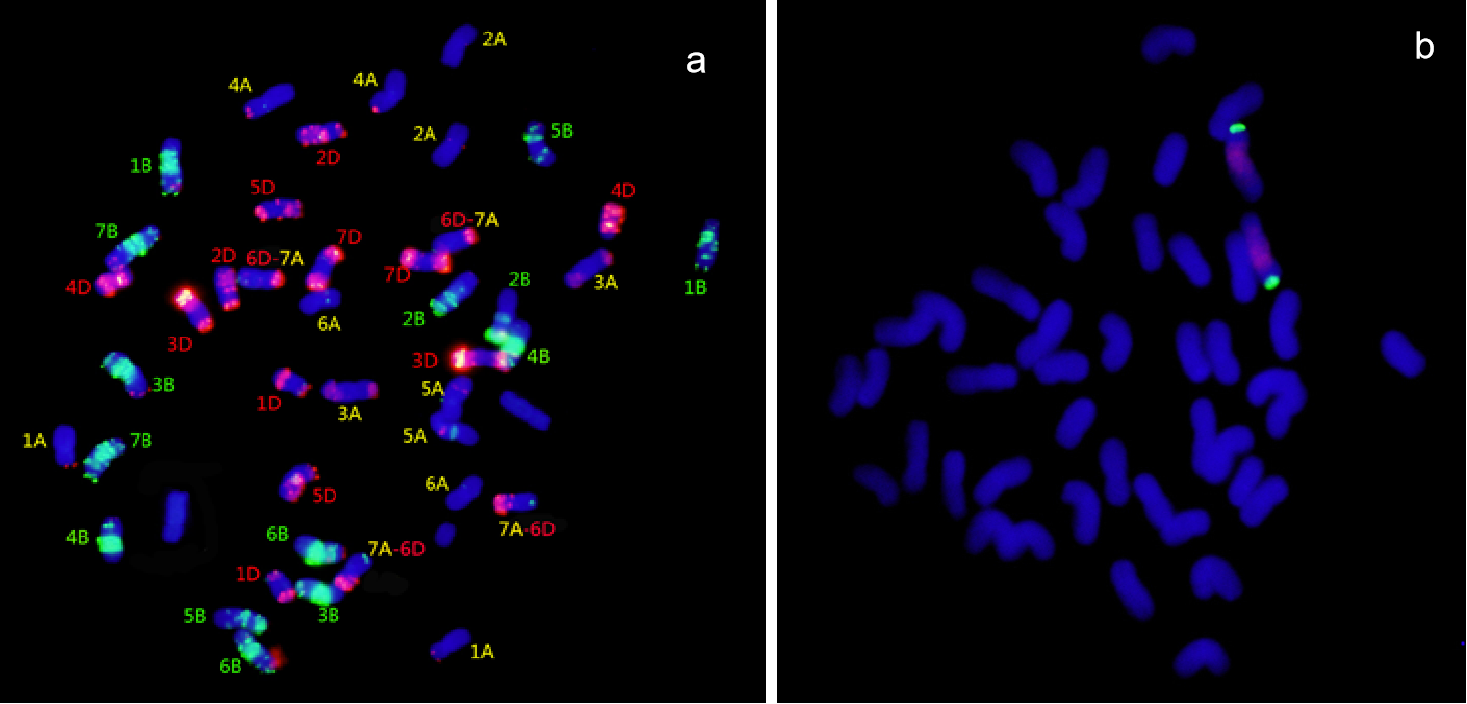


a: pAs1 and pHvG38 were labeled as red and green respectively; b: pAcTRT1 and pAcpCR2 were labeled as red and green respectively, and wheat chromosomes were restained as blue by DAPI.
